# Multi-omics analysis identifies genes mediating the extension of cell walls in the *Arabidopsis thaliana* root elongation zone

**DOI:** 10.3389/fcell.2015.00010

**Published:** 2015-02-20

**Authors:** Michael H. Wilson, Tara J. Holman, Iben Sørensen, Ester Cancho-Sanchez, Darren M. Wells, Ranjan Swarup, J. Paul Knox, William G. T. Willats, Susana Ubeda-Tomás, Michael Holdsworth, Malcolm J. Bennett, Kris Vissenberg, T. Charlie Hodgman

**Affiliations:** ^1^Centre for Plant Integrative Biology, School of Biosciences, University of NottinghamSutton Bonington, UK; ^2^Plant Glycobiology Section, Department of Plant and Environmental Sciences, University of CopenhagenCopenhagen, Denmark; ^3^Centre for Plant Sciences, Faculty of Biological Sciences, University of LeedsLeeds, UK; ^4^Laboratory of Plant Growth and Development, Department of Biology, University of AntwerpAntwerp, Belgium

**Keywords:** root growth, plant cell walls, multiomics, transcriptomics, localisomics, epitomics, cell-wall polysaccharides, cell elongation

## Abstract

Plant cell wall composition is important for regulating growth rates, especially in roots. However, neither analyses of cell wall composition nor transcriptomes on their own can comprehensively reveal which genes and processes are mediating growth and cell elongation rates. This study reveals the benefits of carrying out multiple analyses in combination. Sections of roots from five anatomically and functionally defined zones in *Arabidopsis thaliana* were prepared and divided into three biological replicates. We used glycan microarrays and antibodies to identify the major classes of glycans and glycoproteins present in the cell walls of these sections, and identified the expected decrease in pectin and increase in xylan from the meristematic zone (MS), through the rapid and late elongation zones (REZ, LEZ) to the maturation zone and the rest of the root, including the emerging lateral roots. Other compositional changes included extensin and xyloglucan levels peaking in the REZ and increasing levels of arabinogalactan-proteins (AGP) epitopes from the MS to the LEZ, which remained high through the subsequent mature zones. Immuno-staining using the same antibodies identified the tissue and (sub)cellular localization of many epitopes. Extensins were localized in epidermal and cortex cell walls, while AGP glycans were specific to different tissues from root-hair cells to the stele. The transcriptome analysis found several gene families peaking in the REZ. These included a large family of peroxidases (which produce the reactive oxygen species (ROS) needed for cell expansion), and three xyloglucan endo-transglycosylase/hydrolase genes (XTH17, XTH18, and XTH19). The significance of the latter may be related to a role in breaking and re-joining xyloglucan cross-bridges between cellulose microfibrils, a process which is required for wall expansion. Knockdowns of these XTHs resulted in shorter root lengths, confirming a role of the corresponding proteins in root extension growth.

## Introduction

The plant kingdom displays an enormous diversity in shapes and sizes, varying from unicellular algae with a simple rather spherical morphology to very complex multicellular organisms that can reach more than 100 m in height. Growth of plants is the sum of two processes, namely the increase in cell number by repeated cycles of cell division and the subsequent—sometimes major—increase in volume of these newly formed cells by expansion. Both processes are controlled by the action of plant hormones among which auxin plays a major role (Perrot-Rechenmann, 2010). In roots, cells pass sequentially through different developmental stages along the root axis. Growth occurs through rapid elongation of cells in a zone shootward to the root apical meristem, which is the site of cell division, and before further cell type differentiation, for example root hair emergence and lateral organ initiation (Verbelen et al., 2006).

Plant cell walls are rigid yet deformable materials, and growth is seen as the irreversible increase in surface area of cell walls. This process requires an internal turgor pressure, which arises from water uptake into the cell. Turgor exerts a force against surrounding cell walls, but is otherwise mediated by changes in the mechanical properties of the walls, resulting in stress relaxation (Ray et al., 1972; Cosgrove, 1986, 1993, 2005; Guerriero et al., 2014). Cell walls are made up of a fibrillar component, the cellulose microfibrils, that is embedded in a highly hydrated matrix of pectins, which principally comprise homogalacturonan (HGA), rhamnogalacturonan-I (RG-I), and rhamnogalacturonan-II (RG-II). The tethering of the adjacent cellulose microfibrils occurs primarily through xyloglucan in dicotyledonous and non-commelinid monocotyledonous walls (Hayashi, 1989), or by glucuronoarabinoxylan (Nishitani and Nevins, 1991; Carpita and Gibeaut, 1993) and mixed-linkage (1-3),(1-4)-β-D-glucans in the walls of Poales and Equisetales (Kato et al., 1982; Scheller and Ulvskov, 2010; Mohler et al., 2013).

As well as pectin and hemicelluloses, several different classes of glycoproteins and enzymes are also present (Albenne et al., 2009). This complex composition results in the mechanical properties of the cell wall and greatly influences its growth potential. The volume increase of a plant cell was described in the Lockhart equation (1965), dV/dT = ϕ (P–Y), where (P–Y) is the turgor above a yield threshold Y that must be exceeded before plastic wall extension can occur, and ϕ is the extensibility coefficient that represents the time-dependent yielding properties of the cell wall in the direction of growth (Schopfer, 2006). Several proteins that can influence the cell wall's yielding parameters have been described, including expansins (McQueen-Mason et al., 1992), xyloglucan endotransglucosylase/hydrolases (XET/XTHs; Nishitani and Vissenberg, 2007; Miedes et al., 2013), peroxidases (Passardi et al., 2006), β(1-4)-glucanases (Labrador and Nevins, 1989), yieldins (Okamoto-Nakazato et al., 2001), and lipid transfer proteins (LTPs; Nieuwland et al., 2005).

With the emergence of different molecular biological approaches and tools, many genes that encode enzymes with a role in the synthesis of the various cell wall polysaccharides and proteins found in cell walls have been identified and their mutants described (e.g., Harholt et al., 2010; Carpita, 2011; Mewalal et al., 2014). The synthesis of complete cell-wall components, their trafficking and final assembly in cell walls are, however, very complex (McCann and Rose, 2010) and there is often not a simple link between genotype and a growth phenotype. Furthermore, the content, architecture and biophysical characteristics of the walls of a cell change at any point along the growth axis as a consequence of both the cell's history (i.e., the multiple processes since the cell originated) and the needs of its current location.

As a result of this complexity, point measurements are extremely difficult to interpret and the use of a single “omics” technique to uncover cell-wall processes underpinning plant growth might not be sufficient (Somerville et al., 2004; Farrokhi et al., 2006). Therefore, model organisms, such as *Arabidopsis thaliana*, and appropriate research tools are needed. The *A. thaliana* root has a relatively simple anatomy and develops in a highly predictable manner (Dolan et al., 1993), lending itself to investigation of growth mechanisms, and their regulation as evidenced by numerous reports (e.g., Ubeda-Thomás et al., 2009; Band et al., 2011; Bruex et al., 2012; De Rybel et al., 2012). In addition, its genome sequence is published (Arabidopsis Genome Initiative, 2000) and many research tools already exist (e.g., Fukao et al., 2013; Jacques et al., 2013; Moussaieff et al., 2013).

We used a combination of point measurements and three techniques to characterize the different developmental zones along the *A. thaliana* root, looking at cell wall composition by means of quantitative assessment of cell-wall epitopes (epitomics), epitope localization (localisomics), and gene expression (transcriptomics), and combined the -omics data in this study to provide an integrated perspective. This revealed that individual omics-techniques are inadequate and can even result in misleading conclusions. In contrast, the multi-omics approach has identified three gene families that appear to play a role in regulating root growth, and mutant analysis for one of these families (XTHs) supports these findings.

## Materials and methods

### Plant material and handling

Seeds of *Arabidopsis thaliana* (L.) Heynh. (ecotype Columbia-0) were surface-sterilized by incubation in 5% (v/v) sodium hypochlorite for 5 min, washed three times in sterile water and sown on vertical 125 × 125 mm square Petri plates. Each plate contained 60 ml 1/2 strength Murashige and Skoog media (Sigma) solidified with 1% (w/v) agar. For material used for transcriptomic and glycan microarray profiling (epitomics), sterile 9 × 9 cm square sections of 100 μm nylon mesh (Clarcor) were placed onto the media surface before sowing to facilitate root dissection and harvesting of cut sections. After 2 days at 4°C, plates were transferred to controlled-environment chambers at 23°C under continuous light at a photon flux density of 150 μmol m^−2^ s^−1^ for 7 days.

**Figure 1:**
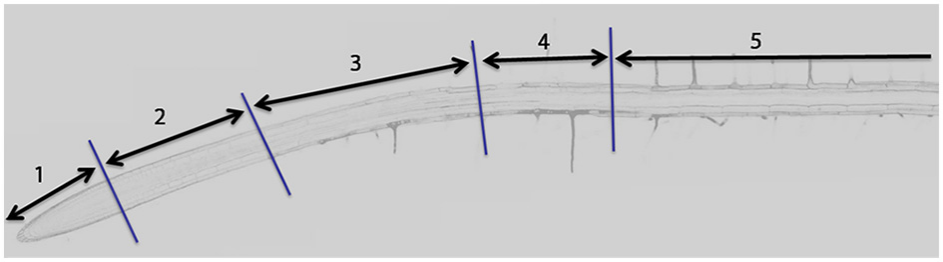
**Overview of the longitudinal sections used for the whole-genome transcript and epitomic analyses**. (1) Meristem (MS); (2) rapid elongation zone (REZ); (3) late elongation zone (LEZ); (4) mature zone (MZ); (5) lateral root zone (LRZ). Image from De Rybel et al. (2010).

Roots were dissected into five sections as shown in Figure 1: (1) meristem (from the root tip to the top of the lateral root cap, approximately 350 μm from the tip); (2) rapid elongation zone (from the top of the lateral root cap to the first visible root hair bulge, approximately 850 μm from the shootward boundary of zone 1); (3) late elongation (deceleration) zone (from the first root hair bulge to the first fully elongated root hair); (4) mature root (500 μm shootward of the first fully elongated root hair); and the lateral root zone (2.5 cm in length, from the shootward boundary of zone 4 in a shootward direction). Dissected samples were immediately frozen in liquid nitrogen.

### Epitomics

Techniques involving glycan microarrays were used for this (Shin et al., 2005; Moller et al., 2007, 2008). Cell wall material was isolated from dissected material as alcohol-insoluble residue (AIR). Frozen material was ground in liquid nitrogen using a micro-pestle, 1 ml of 70% (v/v) ethanol was added to each tube and the mixture shaken at 4°C for 1 h. The mixture was centrifuged (5 min, 10,000 ×*g*) and the supernatant discarded. This process was repeated 4 times. Pellets were resuspended in 1 ml of acetone for 5 min and then air-dried overnight. A total of 600 roots were dissected, yielding 10–20 mg AIR for each of the five sections, which were subjected to three sequential extractions as previously described (Sørensen and Willats, 2011). Briefly, the extractions were performed using 15 μl each of 50 mM diamino-cyclo-hexane-tetra-acetic acid (CDTA), 4 M sodium hydroxide (NaOH) with 0.1% v/v sodium borohydride (NaBH_4_) and cadoxen [31% (v/v) 1,2-diaminoethane with 0.78 M cadmium oxide (CdO)]. These respectively enrich for pectin, hemicelluloses, and cellulose-associated molecules. The extracted fractions were printed as microarrays with six replicates and three dilutions using a Microgrid II microarray robot (Genomic Solutions, Ann Arbor, MI, USA) and the arrays were probed with a range of primary antibodies or carbohydrate-binding modules and appropriate alkaline phosphatase (AP) conjugated secondary antibodies before developing as previously described (Sørensen and Willats, 2011). All JIM- and LM-monoclonal antibodies and 2F4 were obtained from PlantProbes (http://www.plantprobes.net), CCRC antibodies from CarboSource (http://www.ccrc.uga.edu/~carbosource/CSS_home.html) and secondary antibodies from Sigma-Aldrich (http://www.sigmaaldrich.com). The arrays were scanned and analyzed using the microarray software ImaGene 6.0 (http://www.biodiscovery.com) to obtain raw signal values. These were then treated in the same way as fluorescent microarray data. Specifically, the value from the negative control where the secondary antibody was omitted was subtracted, and then the median and median-absolute deviation values were calculated. A heatmap was produced using Microsoft Excel.

### Localisomics

Four-day-old seedlings were fixed and prepared for whole-mount immunolocalization analyses requiring some cell-wall permeabilization steps as described previously (Peret et al., 2012). Cell wall antibodies were used at 1:100 dilutions, whereas Alexafluor488 or Alexafluor543 coupled anti-rat or anti-mouse secondary antibodies were used at 1:200 dilutions to give green or red fluorescence, respectively. Counter staining was performed using either propidium iodide (for the AlexaFluor488 coupled secondary antibody) or Sytox Green (for AlexaFluor543 coupled secondary antibody). Seedlings were mounted in 50% glycerol and images were taken using a Leica SP2 confocal laser scanning microscope (Leica Microsystems UK Ltd). The specific antibodies, their epitopes and localization (primarily as determined in this study) are listed in Table 1.

**Table 1:** **Cell wall epitopes assessed in the glycan microarray and their localization from *in situ* fluorescence studies**.

**Antibody**	**Epitope**	**Localization**	**References/image**
CAL	(1→3) β-glucan	n/a	
CCRC-M1	Xyloglucan	All walls	Figure 3 and Freshour et al., 2003
CBM22	Xylan	Secondary cell walls	McCartney et al., 2006
2F4	Calcium-stabilized homogalacturonan chains	n/a	
LM1	Extensin (HRGP)	No signal	
LM2	AGP (β-linked glucuronic acid)	Mainly lateral root cap and young epidermis; in meristem some cell-wall plates in epidermis and a stele cell file	Supplementary Figure S1, pp. 12–17
LM5	(1→4)-β-D-galactan	All cytoplasm and walls, especially of epidermis and stele	Supplementary Figure S1, p. 18 and McCartney et al., 2003
LM6	(1→5)-α-L-arabinan/AGPs	Epidermis and lateral root cap; higher up root localized to patches possibly forming diagonal stripes	Figure 3 and Talboys et al., 2011
LM8	Xylogalacturonan	Mainly lateral root cap	Supplementary Figure S1, p. 21 and Willats et al., 2008
LM10	(1→4)-β-D-xylan	No signal	
LM15	XXXG motif of xyloglucans	Quiescent center and mature epidermis, especially at interface between cell files and root hairs	Figure 3 and Larsen et al., 2014
JIM5	Partially methylesterified-homogalacturonan	Mainly lateral root cap, walls of quiescent center and initial cells, and some cell-division plates	Figure 3
JIM7	Partially methylesterified-homogalacturonan	No signal	
JIM8	AGP glycan	No signal	
JIM13	AGP glycan	Stele files especially zones 2–5, faint signal in epidermis	Figure 3 and Dolan and Roberts, 1995
JIM19	Extensin	No signal	
JIM20	Extensin	Mainly epidermis and cortex from zone 2 upwards, some in lateral root cap	Figure 3
MAC207	AGP glycan	n/a	

### Transcriptomics

Three biological replicates from separate pools of seeds were used. For each biological replicate, plants were grown and approximately 50 roots dissected as described in Section Plant Material and Handling. RNA was extracted using the Qiagen MicroRNA Kit following the manufacturer's instructions (Qiagen, Crawley, UK) and quantified using a Nanodrop ND100 spectrophotometer (Nanodrop, Wilimington, USA). All RNA samples were approximately 50 ng μl^−1^ in a total volume of 10 μl. Labeling of RNA samples was conducted using the Affymetrix IVT-Express Eukaryotic Target Labeling Assay kits following standard Affymetrix protocols (Affymetrix UK Ltd., High Wycombe, UK). RNA labeling and hybridization to Affymetrix ATH1 arrays were performed by the Nottingham Arabidopsis Stock Centre (NASC).

Data were normalized from.cel files with RMA and statistical tests performed using the Limma package in R/Bioconductor (Smyth, 2005) and a custom CDF file (ATH1121501_At_TAIRT v17; Dai et al., 2005). A gene was considered to be expressed if its expression was greater than 100 and differentially expressed if a *t*-test between two zones was significant at a *q*-value of 0.05 after Benjamini and Hochberg false discovery rate correction (Smyth, 2005). Further analyses were performed using Excel 2010 (Microsoft Corporation, Redmond, USA). Genes were further annotated into cell-wall functional subclasses using Cell Wall Navigator (Girke et al., 2004) and PlnTFDB (Pérez-Rodrłguez et al., 2010). Transcriptomics data used in these experiments have been made available through ArrayExpress (www.ebi.ac.uk) with accession number E-MEXP-2912.

### *xth* mutant analysis

*Atxth17-1*(SALK_015077), *Atxth19-1* (SALK_034274), *Atxth20-1*(SAIL_575_H09), and XTH18-RNAi were kindly provided by Prof. K. Nishitani (Tohoku University, Japan). *Atxth17-2* (SALK_008429), *Atxth19-2* (SAIL_62_A10), and *Atxth20-2* (SALK_066689) were obtained from NASC. All lines are in the Colombia-0 background. To assess basal root growth, the root length of seedlings grown vertically for 7 days was measured from the hypocotyl to the root tip. Root lengths were measured using the NeuronJ plugin of ImageJ 1.4.1j (http://rsb.info.nih.gov/ij/). Two-tail Student *t*-tests were performed using Excel 2010 to determine significance (*p*-value < 0.05).

Confocal microscopy for imaging of *A. thaliana* roots was performed using a Leica SP5 confocal laser-scanning microscope (Leica, Milton-Keynes, UK). For cell quantification and cell length measurements, seedlings were treated with propidium iodide (10 μg ml^−1^; Sigma) to visualize cell walls. Cell lengths were measured using the Cell-o-Tape image-analysis tool (French et al., 2012). Data are presented as the mean ± the standard error and two-tail Student *t*-tests, used to determine significance (*p*-value < 0.05), were performed using Microsoft Excel software.

## Results

### Epitomics

**Figure 2:**
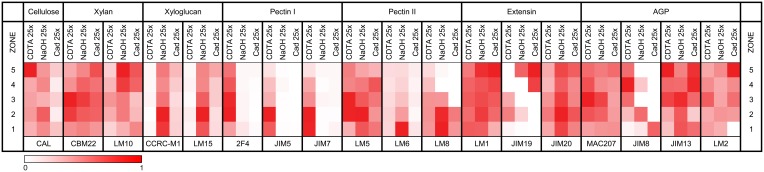
**Epitomic heatmap**. The results for each antibody are scaled relative to the maximum signal for that antibody. The heatmap is supplemented by Supplementary Figure S2, which shows the actual signals in graph form for each antibody. Pectin I corresponds to homogalacturonan epitopes and Pectin II to rhamnogalacturonans.

Figure 2 depicts the epitope intensities in the different fractions and root zones, and highlights the zonal difference between antibodies to related epitopes. The signals corresponding to the pectin I and xyloglucan (XyG) binding antibodies, suggests that the CDTA and NaOH treatments were effective in terms of extracting the associated polysaccharides (pectin and hemicelluloses, respectively), since it was expected that some cellulose-associated XyG would be extracted using cadoxen due to the fact that XyG tethers adjacent cellulose microfibrils.

The more soluble (1→3)-β-glucans, recognized by CAL, peaked in zones 2 and 4 in the hemicellulosic fraction while in the pectin fraction they actually peaked in zone 5, consistent with the highest pectin-associated signal of crystalline cellulose. The xylans (recognized by LM10 and CBM22) were present in all three fractions. The CBM22 signal peaked in zones 1–3 in the pectin fraction, in zones 2–4 in the hemicellulosic fraction and in zone 5 in the cellulosic fraction. LM10, however, showed an increasing signal in the hemicellulosic fraction as the root matured, a trend that was also present in the cellulosic fraction where it was accompanied by a reduction in zones 2 and 3.

Xyloglucans (probed by CCRC-M1 and LM15) peaked in the REZ and were specific to the hemicellulosic fraction, with only minor binding in the cellulosic fraction, a pattern becoming more evident as the root matures. The homogalacturonan/pectin I epitope, probed by 2F4, JIM5, and JIM7, which differ in their sensitivity to the degree of esterification, shows a similar pattern and is specific to the pectin fraction. JIM7 peaked in zones 1 and 2, JIM5 peaked in zone 2, and 2F4 peaked in zones 2 and 3.

The pectin II epitopes show a broad range of patterns, consistent with the complexity of its polymer constituents. LM6, specific for arabinan, peaked in the hemicellulosic fraction in zone 1 and rapidly decreased as the root matures. LM8, specific for xylogalacturonan, peaked in zones 1 and 2 in the same fraction and to a lesser extent in the cellulosic fraction before trailing off rapidly after zone 3, whilst in the pectin fraction it was consistently present at a low level throughout. LM5, recognizing galactan, shows one of the most complex signal patterns, peaking in zones 2–3 in the pectin fraction, zone 2 in the hemicellulosic fraction while showing the inverse pattern in the cellulosic fraction.

Three antibodies detect variants from the extensin family. The LM1 and JIM20 epitopes are present at high levels in all zones, with the JIM20 signal being higher in the hemicellulosic fraction, peaking in zone 2, and the LM1 signal being nominally highest in zone 5. The JIM19 epitope, however, was absent from the meristem, was mostly pectin-associated in the elongation zones, and showed its highest signal in zones 4 and 5, but was specifically non-pectin associated. Several antibodies recognize different epitopes associated with AGPs. Their signal levels tended to be higher from zone 2 onwards, but they were also found in all extractions and developmental zones.

### Localisomics

**Figure 3:**
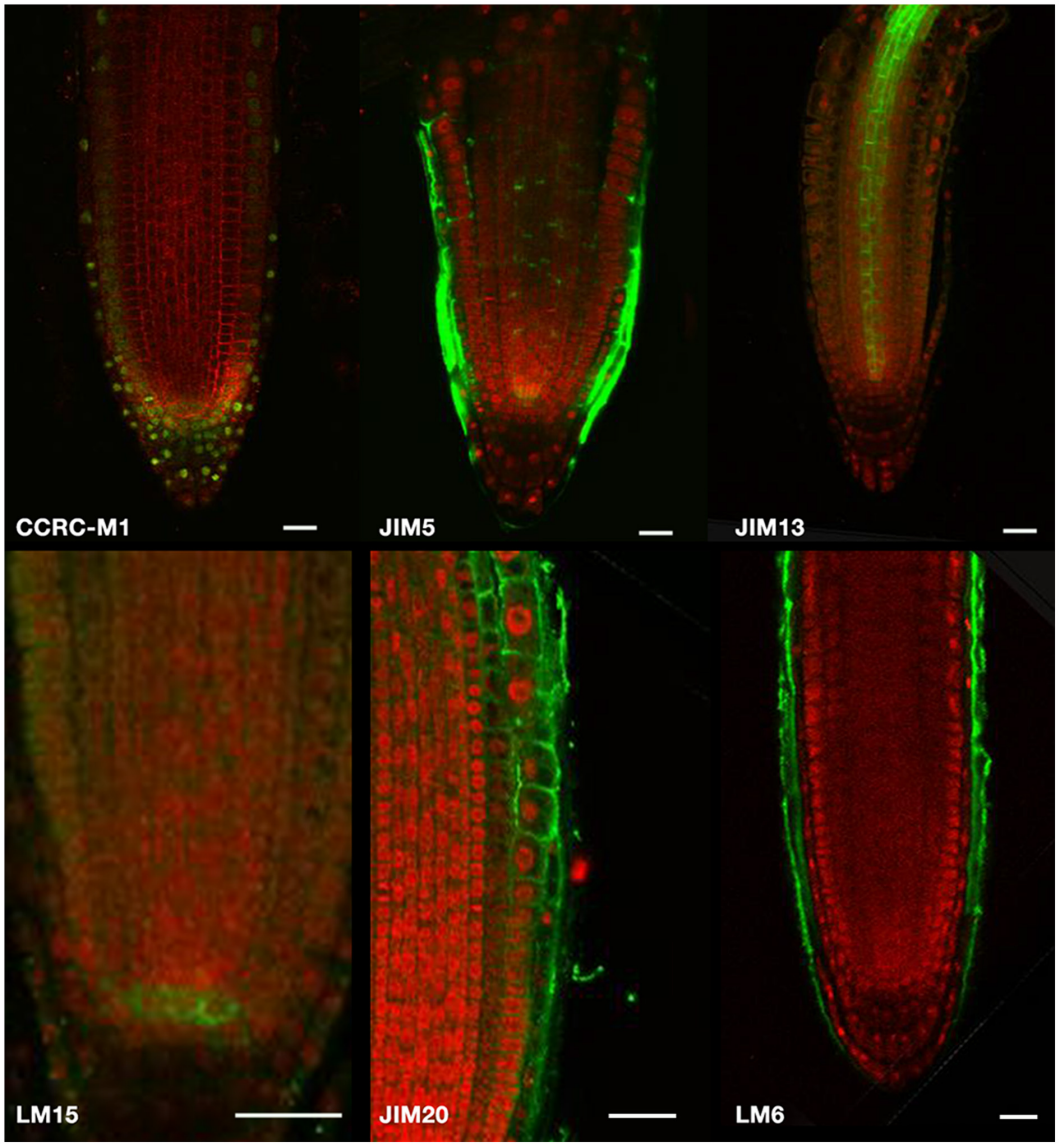
**Immunolocalization with key antibodies**. CCRC-M1 and LM15 detect xyloglucans, JIM5 detects pectins, JIM20 detects extensins, and JIM13 and LM6 bind to AGPs. An AF488-linked secondary antibody was used for all antibodies except CCRC-M1 for which an AF543-linked antibody was used. The scale bars correspond to 100 μm for all images except LM15 for which it is 25 μm.

Figure 3 contains localization results for four classes of cell wall-related epitopes, namely XyGs, pectins, AGPs, and extensins. Crucially, the images show that antibodies were able to bind to epitopes throughout the root, rather than only at the surface, giving us confidence that cell-wall epitope localization can be obtained when using permeabilization procedures that will lead to the loss of some cell wall structures. However, five antibodies were not available for localization studies and a further five gave no signal, possibly due to the epitope being modified beyond recognition by the permeabilization process. The full image dataset can be found in the Supplementary Material (Supplementary Figure S1) and epitope localization arising from this dataset is described in Table 1.

The XyGs recognized by LM15 were present at low levels throughout, but with a particularly high signal in the quiescent center, although no such specificity was seen with the CCRC-M1 antibody, recognizing fucosylated XyG. JIM5 showed the partially methylesterified pectins to be localized to the lateral root cap and radial walls in the inner tissues, while the extensins detected by JIM20 were most prominent in the epidermis and cortex of the REZ and to a lesser degree in the lateral root cap. JIM13 and LM6 binding patterns suggested that individual AGPs might be specific to different locations, suggesting that they play specific roles in the cells where they are expressed but that their general role in root growth may be difficult to interpret. Finally, binding of the LM8 antibody, recognizing xylogalacturonan, was restricted to the lateral root cap in a manner reminiscent of LM6 (see Supplementary Material).

### Transcriptomics

A large percentage of the genome is expressed in the root with about 7500–8000 genes detectably expressed in any given root zone (See Table 2 and Supplementary Table 1). Zones 1–3, despite their physical proximity, show large inter-zonal differences consistent with their very different developmental roles. Zones 3–5 have more similar gene expression levels, highlighting the general developmental quiescence of the mature root. However, ~10% of significantly differentially expressed genes in these zones are annotated as cell-wall genes, which is twice the number of genes changing between the earlier zones. It is the changes between zones 1–2 and 2–3 that are likely to inform the expression changes that allow rapid elongation and deceleration, respectively. At their peak, 52% of reactive oxygen species (ROS) and 63% of aquaporin genes are expressed in the root. Only 25% of annotated transcription factors are detectably expressed in the root, which is lower than other annotated gene families—possibly reflecting tight developmental differentiation.

**Table 2:** **Transcriptomic data for the five zones of the *A. thaliana* root**.

	**Genes expressed (% of 21,331)**	**Significantly up-regulated**	**Significantly down-regulated**
Zone 1	7741 (36.3%)		
Zone 1–2		1632	1939
Zone 2	7539 (35.3%)		
Zone 2–3		1343	1750
Zone 3	7801 (36.6%)		
Zone 3–4		155	369
Zone 4	7854 (36.8%)		
Zone 4–5		155	184
Zone 5	8044 (37.7%)		
Zone 5–1		1322	2627

*A gene was considered to be expressed if its expression was greater than 100 and differentially expressed if a t-test between two zones was significant at a q-value of 0.05*.

## Discussion

The *A. thaliana* root develops in a highly predictable manner. Cells pass through consecutive developmental phases during which the post-mitotic elongation of cells contributes the majority of the increase in the root length. Cell wall metabolism is very important in allowing and controlling cellular expansion. The synthesis, trafficking, deposition, integration, and remodeling pathways of cell-wall components are, however, very complex and not completely resolved, so multiple omics-approaches are needed to establish which genes are contributing to the observed changes in composition and consequent mechanical properties. These issues are complicated further by the observation that the different tissues differentially contribute to the mechanics of root elongation (Dyson et al., 2014).

### Epitomics analysis

Epitomics (also known as glycan microarray analysis and previously described as Comprehensive Microarray Polymer Profiling) is a high-throughput microarray-derived technique that allows the handling of multiple samples. In this study, it was used to indicate which polysaccharides and glycoproteins were found in specific root developmental-zones. Many of the results support current knowledge of root cell-wall composition, for example that pectin is synthesized and deposited into the existing cell wall in a highly esterified form (Liners and Van Cutsem, 1992) and that pectinesterases modify the pectins while the cells age (Micheli, 2001). Carbohydrate polymer synthesis is complicated and does not necessarily correlate well with wall composition. Little is known of extant synthesis pathways, let alone of any differences in synthesis along developmental zones. In addition, the spatial resolution of this experimental approach is rather poor.

### Localisomics analysis

In contrast to epitomics, whole-mount immunolocalization provides high spatial resolution and revealed cell and tissue-specific locations for some cell wall epitopes that would not have been clear from the epitomic data alone. The AGPs appear to be the most remarkable in this respect, suggesting that individual proteins play a specific and subtly different role. The localization of extensins to zone 2 epidermis and cortex is intriguing, given the predictions of Dyson et al. (2014) that the outer layers of the root have most influence on growth. Unfortunately, some of the antibodies were not available for this technique and others did not yield any signal in the cell walls, probably because of masking of the epitope by other cell wall components or more likely due to the use of enzymes in the permeabilization procedures required for whole-mount preparations. This -omics has refined the roles of certain epitopes, but cannot be used to link to specific genes.

### Transcriptomics analysis

Analysis of the transcriptomic data in isolation can also be misleading. Three gene families account for nearly as much of the expression as all the other cell-wall-related families together. The largest cell wall family is the AGPs with 61 members representing more than 18% of cell wall-related gene expression, but the role of individual genes in expansion and maturation is unclear as the localization shows that different epitopes are found in different places.

Aquaporins play a role in vacuolar filling and contribute to turgor pressure, the driving force of expansion. Their expression is present in the elongation zone (zones 2 and 3), naively suggesting that expansion is effected by an increase in turgor pressure. However, recent work (Dyson et al., 2014) shows that pressures remain constant, implying that the role of the aquaporins is to ensure that turgor pressure is not lost by the rapid expansion in cell size.

The third highly-expressed gene family encodes peroxidases, which contains members that are involved in the generation of ROS. These have long been implicated in root growth and development (Gapper and Dolan, 2006; Manzano et al., 2014). However, our data (Supplementary Table 1) show that their expression rises in zone 2, peak in zone 3 and remain high in the later zones. This suggests a role in maturation rather than elongation, and also possibly in lignification and Casparian strip formation. Although the other -omics data indicated a role for extensins, the genes for these proteins are difficult to define because sequences/functions overlap with other gene families. Hence they were not considered further in this context. Initial carbohydrate biosynthesis peaks in zone 2, where large amounts are needed for both accelerating and subsequent decelerating expansion, but in order to get enough RNA these transcriptomic data face the same issue of resolution as the epitomics work.

### Multi-omics analysis

As mentioned above, all these techniques in isolation have their benefits and drawbacks. We therefore combined data from all analysis in a multi-omics approach to identify genes that play an important role in the elongation of *A. thaliana* root cells.

**Figure 4:**
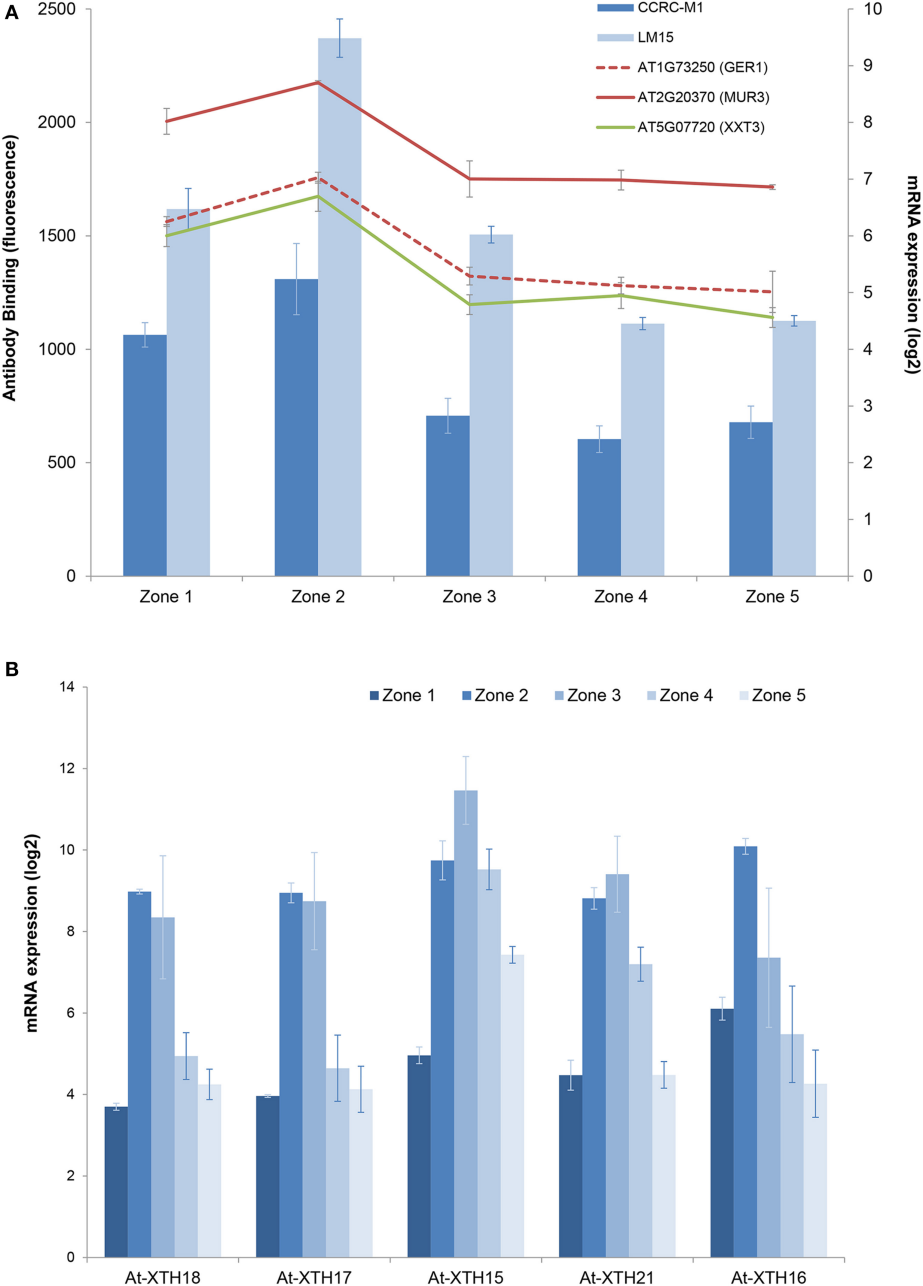
**Combined xyloglucan, XyG biosynthesis genes, and XTH expression profiles. (A)** epitomic (columns) and XyG biosynthesis mRNA expression (lines). **(B)** zonal transcriptomic expression profiles for selected members of the XTH family. Error bars are ±1 SD.

To investigate xyloglucans, we looked for expression patterns in the transcriptome that correlated with the epitope pattern shown by LM15 (peaking in the zone 2). The transcriptomic dataset showed a large number of genes with a similar pattern (Supplementary Table 1). However, filtering the data to include only genes known to affect XyG biosynthesis, we found genes involved in fucose biosynthesis (*MUR3*, AT2G20370 and *GER1*, AT1G73250) and a xyloglucan xylosyltransferase, (*XXT3*, AT5G07720) with *R*^2^-values of 0.96–0.98, suggesting that these members from large gene families may be responsible for the observed LM15-XyG signal (Figure 4).

XyG is highest in zone 2, the rapid elongation zone, correlating with studies showing that *in vivo*, XET activity by XTHs is also highest in this zone (Vissenberg et al., 2000). The general XTH expression signal peaks in zone 2 and 3 (Figure 4), suggesting an important role for XTHs in root elongation. XTHs remodel the cell wall and some are believed to promote growth acceleration (Van Sandt et al., 2007), while others could aid in the deceleration. Different family members have distinct activity dependencies and pH optima, suggesting that some can act as loosening factors, while others could do the opposite (Maris et al., 2009, 2011). If they were only associated with accelerated cell wall actions, then the plant might override the loosening in the deceleration zone, probably by pectin modifications (Micheli, 2001) or peroxidase-mediated cross-linking of other cell-wall components such as structural proteins (Ma et al., 2004; Passardi et al., 2004; De Cnodder et al., 2005). The key XTHs controlling expansion might be expected to be expressed as early as possible to tightly regulate wall extensibility and disruption of these XTHs may therefore show growth defects.

Looking for XTHs with a peak very early in root development and associated with gibberellic acid (GA), a known regulator of cell expansion (Middleton et al., 2012), showed that XTH17 and 18 increase more than 30 fold between zones 1 and 2. One of these is GA-induced (XTH17) and both belong to a subclade of group 1 XTHs (Rose et al., 2002) consisting of 4 genes (XTH17, 18, 19, and 20). XTH17, 18, and 19 are expressed in the elongation zones of the root, and XTH20 is expressed in the vascular tissue of the mature root (Vissenberg et al., 2005). We therefore investigated phenotypes in mutant lines for this subclade. Putative knock out (KO) lines were identified for three of the genes and an RNAi line was created for the fourth. All these lines showed significant growth defects with shorter mature root lengths, shorter mature root cells and reduced growth rates (Tables 3, 4). The one exception to this was *xth19-1* which, in contrast to the *xth19-2* line, showed an increase in root length. The insertion in the *xth19-1* line is 3′ to the gene, which may stabilize the mRNA and hence act as an over-expression line. The reduction in growth phenotype was increased in the *xth17-2*xXTH18-RNAi double mutant (Table 4).

**Table 3:** **Mean root lengths in wild type and *xth* mutants, measured at 7 days after germination, asterisks denote significance at a *p* > 0.05**.

	**Length (mm ± *SEM*)**	**% vs. Col-0**	***n***
**SINGLE MUTANTS**			
Col-0	37.87 (±0.39)	–	71
*xth17-1*	34.01 (±0.40)^*^	89.8%	44
*xth17-2*	34.09 (±0.46)^*^	90.0%	39
xth18-RNAi	32.75 (±0.65)^*^	86.5%	26
*xth18-2*	38.74 (±0.45)	102.3%	40
*xth19-1*	41.31 (±0.29)^*^	109.1%	47
*xth19-2*	32.36 (±0.48)^*^	85.5%	19
*xth20-1*	35.17 (±0.48)^*^	92.9%	41
*xth20-2*	33.73 (±0.57)^*^	89.1%	36
**DOUBLE MUTANTS**			
Col-0	38.02 (±0.39)	–	69
*17-1*×*18-1*	32.09 (±0.60)^*^	84.4%	25
*17-2*×*18-1*	27.77 (±0.61)^*^	73.0%	31
*17-2*×*19-1*	33.63 (±0.54)^*^	88.4%	25
*19-1*×*20-2*	34.02 (±1.21)^*^	89.5%	20
*17-1*×*20-1*	35.29 (±0.40)^*^	92.8%	40
*18-1*×*20-1*	36.13 (±0.73)^*^	95.0%	24

**Table 4:** **Effect of reduced XTH17 and XTH18 expression on root growth**.

	**Growth rate (mm/h)**					**Average mature cell length (μm)**
	**3 dag**	**4 dag**	**5 dag**	**6 dag**	**7 dag**	
Col-0	0.086 (±0.003)	0.219 (±0.013)	0.314 (±0.018)	0.370 (±0.013)	0.407 (±0.021)	199.18 (±1.10)
*xth17-2*	0.075 (±0.006)^*^	0.206 (±0.016)	0.255 (±0.024)^*^	0.354 (±0.026)	0.367 (±0.030)	184.42 (±7.88)^*^
XTH18-RNAi	0.070 (±0.005)^*^	0.172 (±0.016)^*^	0.286 (±0.020)	0.359 (±0.015)	0.357 (±0.016)^*^	189.66 (±6.78)^*^
*xth17-2* × XTH18-RNAi	0.054 (±0.004)^*^	0.193 (±0.017)^*^	0.240 (±0.025)^*^	0.241 (±0.019)^*^	0.319 (±0.052)^*^	182.06 (±7.66)^*^

*Growth rates were measured at the indicated days after germination (dag) using NeuronJ. Cortical cell lengths were measured from confocal microscope images, asterisks denote significance at a p > 0.05*.

**Figure 5:**
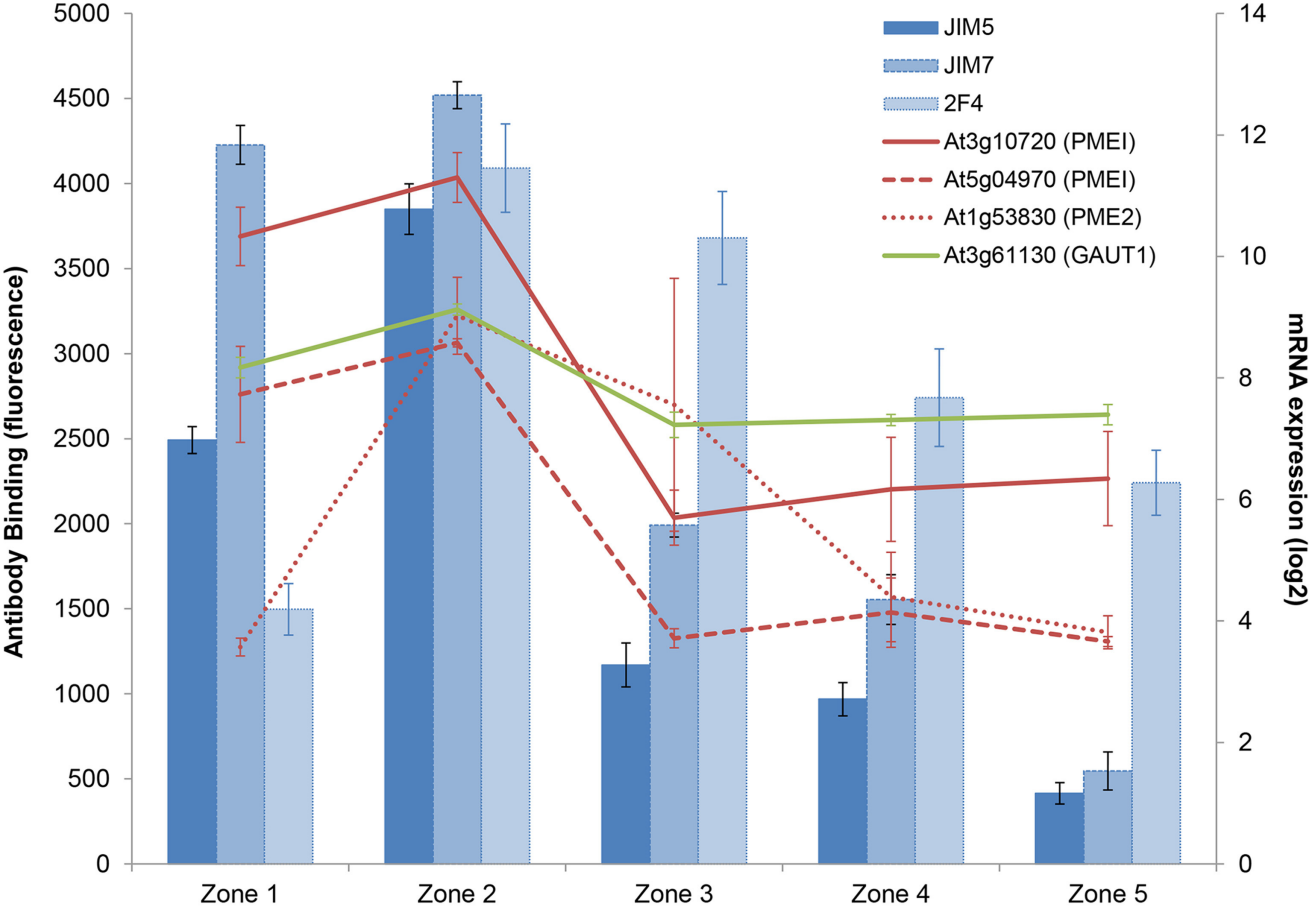
**Combined profiles of pectin epitopes, GAUT1, PME, and PMEI expressions**. Epitomic (columns) and biosynthesis (GAUT1) and modification (PME and PMEI) mRNA expression (lines). Error bars are ±1 SD.

Regarding pectin (or more specifically homogalacturonan), JIM5, and JIM7 recognize esterified pectin, which is present at highest levels in zones 1 and 2 and drops off in the mature root. Pectin is deposited into the cell wall in a highly esterified form, and is typically a component of a loosened cell wall. 2F4 recognizes cross-linked pectin with no more than 40% esterification (Liners et al., 1992) and this is low in zone 1 but rises to high levels in zone 2 and beyond. This suggests zone 2 has a mixture of esterified and non-esterified forms and the distribution is increasingly biased toward non-esterified along the shootward axis. This follows the expression of several pectin methylesterases [PMEs; e.g., PME2 (AT1G53830)], some of which are highly correlated with the epitomic pattern (Figure 5).

Several PME Inhibitors (e.g., AT5G04970, AT3G10720) also correlate with this pattern (Figure 5), suggesting that the root uses a tight balance of these two groups of enzymes to control the rate of de-esterification (i.e., stiffening) rather than using one to shut the other off. With regard to pectin biosynthesis, the galacturonosyltransferase 1 enzyme (GAUT1, At3g61130) is expressed in zones 1 and 2 and then decreases, which correlates (*R*^2^ > 0.9) with the pattern of the three antibody epitopes recognizing homogalacturonan (Figure 5 and Supplementary Figure S2).

**Figure 6:**
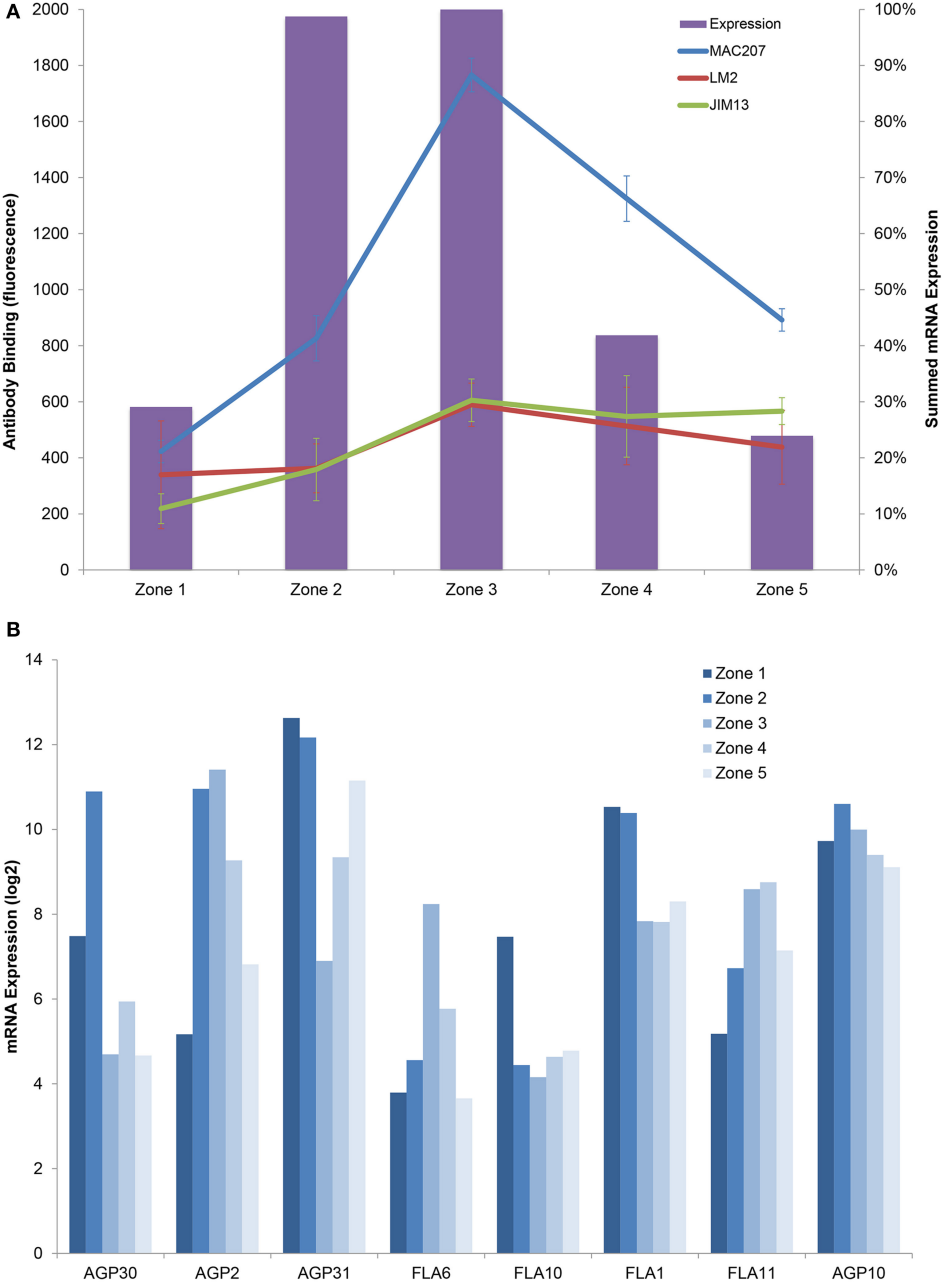
**Combined profiles of AGP epitopes and expression. (A)** epitomic (lines) and mRNA expression (columns), expression is summed over all expressed AGPs and given as a percentage of the expression in each zone, error bars are ±1 SD. **(B)** Exemplars of the principal expression profiles for members of the AGP family.

The transcriptomics data revealed multiple patterns for AGP expression, dominated by maximum levels in zones 2 and 3 (Figure 6). In contrast, the epitomic signals peak in zone 3, which could be accounted for by several hypotheses. One possibility is that there are delays in synthesis and transport either because of long synthesis and transport times of the proteins, or accumulation for use in zone 3 walls. Alternatively the epitomic profiles could simply be reflecting AGP accumulation over time.

Different AGPs may play different roles in the cell wall as revealed by the localisome, which was also mentioned before (Ellis et al., 2010). For example, LM6 (AGP or pectic arabinan) localizes to the lateral root cap and epidermis and its epitope may play a role in expansion, while the JIM13 epitope is specific to stele cell files and could be part of the process of vascular patterning.

### Overarching conclusions

It is evident that the individual -omics approaches provide an incomplete picture, and a combination of multiple analyses aids in establishing a clearer picture of the processes involved. In general terms, the transcriptomic dataset suggests the location of cell wall synthesis, whereas the glycan microarray analyses show the accumulation and dilution as these polymers are modified or additional material added. This is best shown with regard to pectins and XyGs. Deceleration in the root elongation rate appears to be linked to a change in the ratio of esterified to non-esterified pectins. The mutation studies have confirmed a role for XTH17, XTH18, and possibly XTH19 in root growth, probably affecting yield threshold, as this was the case in dark-grown hypocotyl cells (Miedes et al., 2013).

### Potential application to other systems

Root growth is a system in which the genes for synthesis of cell wall material are expressed in one location, while the molecules themselves might not appear in the wall until later. Different molecules contribute to different aspects of the cell-wall mechanics, which are further affected by subsequent modification and interaction. The multi-omics approach used in this study could be used for other plant structures and translated to other systems where the chronology of gene expression, macromolecular synthesis, and modification contribute to growth or mechanical properties of an organ as a whole. Potential applications include musculoskeletal growth, strength, and brittleness in health, aging and disease states, as well as plant lodging (i.e., the bending or even falling over of stalks leading to reduced crop yields).

### Conflict of interest statement

The authors declare that the research was conducted in the absence of any commercial or financial relationships that could be construed as a potential conflict of interest.

## References

[B1] Albenne C. Canut H. Boudart G. Zhang Y. San Clemente H. Pont-Lezica R.. (2009). Plant cell wall proteomics: mass spectrometry data, a trove for research on protein structure/function relationships. Mol. Plant. 2, 977–989. 10.1093/mp/ssp059 19825673

[B2] Arabidopsis Genome Initiative. (2000). Analysis of the genome sequence of the flowering plant *Arabidopsis thaliana*. Nature 408, 796–815. 10.1038/35048692 11130711

[B3] Band L. R. Wells D. M. Larrieu A. Sun J. Middleton A. M. French A. P. (2011). Root gravitropism is regulated by a transient lateral auxin gradient controlled by a tipping-point mechanism. Proc. Natl. Acad. Sci. U.S.A. 109, 4668–4673 10.1073/pnas.1201498109 22393022 PMC3311388

[B4] Bruex A. Kainkaryam R. M. Wieckowksi Y. Kang Y. H. Bernhardt C. Xia Y.. (2012). A gene regulatory network for root epidermis cell differentiation in Arabidopsis. PLOS Genet. 8:e1002446. 10.1371/journal.pgen.1002446 22253603 PMC3257299

[B5] Carpita N. C. (2011). Update on mechanisms of plant cell wall biosynthesis: how plants make cellulose and other (1→4)-β-d-glycans. Plant Physiol. 155, 171–184. 10.1104/pp.110.163360 21051553 PMC3075763

[B6] Carpita N. C. Gibeaut D. M. (1993). Structural models of primary cell walls in flowering plants: consistency of molecular structure with the physical properties of the walls during growth. Plant J. 3, 1–30. 10.1111/j.1365-313X.1993.tb00007.x 8401598

[B7] Cosgrove D. J. (1986). Biophysical control of plant cell growth. Annu. Rev. Plant Physiol. 37, 377–405. 10.1146/annurev.pp.37.060186.002113 11539701

[B8] Cosgrove D. J. (1993). Water uptake by growing cells: an assessment of the controlling roles of wall relaxation, solute uptake, and hydraulic conductance. Int. J. Plant Sci. 154, 10–21. 10.1086/297087 11537965

[B9] Cosgrove D. J. (2005). Growth of the plant cell wall. Nat. Rev. Mol. Cell Biol. 6, 850–861. 10.1038/nrm1746 16261190

[B10] Dai M. Wang P. Boyd A. D. Kostov G. Athey B. Jones E. G.. (2005). Evolving gene/transcript definitions significantly alter the interpretation of GeneChip data. Nucleic Acids Res. 15, e175 10.1093/nar/gni179 16284200 PMC1283542

[B11] De Cnodder T. Vissenberg K. Van Der Straeten D. Verbelen J.-P. (2005). Regulation of cell length in the *Arabidopsis thaliana* root by the ethylene precursor 1-aminocyclopropane-1-carboxylic acid: a matter of apoplastic reactions. New Phytol. 168, 541–550. 10.1111/j.1469-8137.2005.01540.x 16313637

[B12a] De Rybel B. Audenaert D. Xuan W. Overvoorde P. Strader L. C. Kepinski S.. (2012). A role for the root cap in root branching revealed by the non-auxin probe naxillin. Nat. Chem. Biol. 8, 798–805. 10.1038/nchembio.1044 22885787 PMC3735367

[B12] De Rybel B. Vassileva V. Parizot B. Demeulenaere M. Grunewald W. Audenaert D.. (2010). A novel aux/IAA28 signaling cascade activates GATA23-dependent specification of lateral root founder cell identity. Curr. Biol. 20, 1697–1706. 10.1016/j.cub.2010.09.007 20888232

[B13] Dolan L. Janmaat K. Willemsen V. Linstead P. Poethig S. Roberts K.. (1993). Cellular organisation of the *Arabidopsis thaliana* root. Development 119, 71–84. 8275865 10.1242/dev.119.1.71

[B14] Dolan L. Roberts K. (1995). Secondary thickening in roots of *Arabidopsis thaliana*: anatomy and cell surface changes. New Phytol. 131, 121–128 10.1111/j.1469-8137.1995.tb03061.x 33863160

[B15] Dyson R. J. Vizcay-Barrena G. Band L. R. Fernandes A. N. French A. P. Fozard J. A.. (2014). Mechanical modelling quantifies the functional importance of outer tissue layers during root elongation and bending. New Phytol. 202, 1212–1222. 10.1111/nph.12764 24641449 PMC4286105

[B16] Ellis M. Egelund J. Schultz C. J. Bacic A. (2010). Arabinogalactan-proteins: key regulators at the cell surface? Plant Physiol. 153, 403–419. 10.1104/pp.110.156000 20388666 PMC2879789

[B17] Farrokhi N. Burton R. A. Brownfield L. Hrmova M. Wilson S. M. Bacic A.. (2006). Plant cell wall biosynthesis: genetic, biochemical and functional genomics approaches to the identification of key genes. Plant Biotechnol. J. 4, 145–167. 10.1111/j.1467-7652.2005.00169.x 17177793

[B18] French A. P. Wilson M. H. Kenobi K. Dietrich D. Voß U. Ubeda-Tomás S.. (2012). Identifying biological landmarks using a novel cell measuring image analysis tool: Cell-o-Tape. Plant Methods 8:7. 10.1186/1746-4811-8-7 22385537 PMC3359173

[B19] Freshour G. Bonin C. P. Reiter W. D. Albersheim P. Darvill A. G. Hahn M. G. (2003). Distribution of fucose-containing xyloglucans in cell walls of the mur1 mutant of Arabidopsis. Plant Physiol. 131, 1602–1612. 10.1104/pp.102.016444 12692319 PMC166916

[B20] Fukao Y. Yoshida M. Kurata R. Kobayashi M. Nakanishi M. Fujiwara M.. (2013). Peptide separation methodologies for in-depth proteomics in Arabidopsis. Plant Cell Physiol. 54, 808–815. 10.1093/pcp/pct033 23426071

[B21] Gapper C. Dolan L. (2006). Control of plant development by reactive oxygen species. Plant Physiol. 141, 341–345. 10.1104/pp.106.079079 16760485 PMC1475470

[B22] Girke T. Lauricha J. Tran H. Keegstra K. Raikhel N. (2004). The cell wall navigator database. A systems-based approach to organism-unrestricted mining of protein families involved in cell wall metabolism. Plant Physiol. 136, 3003–3008. 10.1104/pp.104.049965 15489283 PMC523362

[B23] Guerriero G. Hausman J.-F. Cai G. (2014). No stress! Relax! Mechanisms governing growth and shape in plant cells. Int. J. Mol. Sci. 15, 5094–5114 10.3390/ijms15035094 24663059 PMC3975442

[B24] Harholt J. Suttangkakul A. Scheller H. V. (2010). Biosynthesis of pectin. Plant Physiol. 153, 384–395. 10.1104/pp.110.156588 20427466 PMC2879803

[B25] Hayashi T. (1989). Xyloglucans in the primary cell wall. Annu. Rev. Plant Physiol. Plant Mol. Biol. 40, 139–168 10.1146/annurev.pp.40.060189.001035

[B26] Jacques E. Buytaert J. Wells D. M. Lewandowski M. Bennett M. J. Dirckx J.. (2013). MicroFilament Analyzer, an image analysis tool for quantifying fibrillar orientation, reveals changes in microtubule organization during gravitropism. Plant J. 74, 1045–1058. 10.1111/tpj.12174 23489480

[B27] Kato Y. Ito S. Iki K. Matsuda K. (1982). Xyloglucan and *r*β-D-glucan in cell walls of rice seedlings. Plant Cell Physiol. 23, 351–364.

[B28] Labrador E. Nevins D. J. (1989). An exo-/β-D-glucan derived from Zea coleoptile walls with a capacity to elicit cell elongation. Physiol. Plant. 77, 479–486 10.1111/j.1399-3054.1989.tb05380.x

[B29] Larsen E. R. Domzych D. S. Tierney M. L. (2014). SNARE VTI13 plays a unique role in endosomal trafficking pathways associated with the vauole and is essential for cell wall organization and root hair growth in arabidopsis. Ann. Bot. 114, 1147–1159. 10.1093/aob/mcu041 24737717 PMC4195547

[B30] Liners F. Thibault J.-F. Van Cutsem P. (1992). Influence of the degree of polymerization of oligogalacturonates and of esterification pattern of pectin on their recognition by monoclonal antibodies. Plant Physiol. 99, 1099–1104. 10.1104/pp.99.3.1099 16668976 PMC1080589

[B31] Liners F. Van Cutsem P. (1992). Distribution of pectic polysaccharides throughout walls of suspension-cultured carrot cells. Protoplasma 170, 10–21 10.1007/BF01384453

[B32] Lockhart J. A. (1965). An analysis of irreversible plant cell growth. J. Theor. Biol. 8, 264–275 10.1016/0022-5193(65)90077-9 5876240

[B33] Ma H. Tan L. Kamyab A. Hare M. Shpak E. Kieliszewski M. J. (2004). Di-isodityrosine is the intermolecular cross-link of isodityrosine-rich extensin analogs cross-linked *in vitro*. J. Biol. Chem. 279, 55474–55482. 10.1074/jbc.M408396200 15465824

[B34] Manzano C. Pallero-Baena M. Casimiro I. De Rybel B. Orman-Ligeza B. Van Isterdael G. (2014). The emerging roles of ROS signalling during lateral root development. Plant Physiol. 30, 1105–1119 10.1104/pp.114.238873 24879433 PMC4081325

[B35] Maris A. Kaewthai N. Eklöf J. M. Miller J. G. Brumer H. Fry S. C. (2011). Characterization of five recombinant xyloglucan endotransglucosylase/hydrolase (XTH) proteins of Arabidopsis reveals specific enzymatic properties. J. Exp. Bot. 62, 261–271 10.1093/jxb/erq263 20732879

[B36] Maris A. Suslov D. Fry S. C. Verbelen J.-P. Vissenberg K. (2009). Enzymic characterization of two recombinant xyloglucan endotransglucosylase/hydrolase (XTH) proteins of Arabidopsis and their effect on root growth and cell wall extension. J. Exp. Bot. 60, 3959–3972. 10.1093/jxb/erp229 19635745

[B37] McCann M. Rose J. (2010). Blueprints for building plant cell walls. Plant Physiol. 153, 365. 10.1104/pp.110.900324 20522725 PMC2879777

[B38a] McCartney L. Blake A. W. Flint J. Bolam D. N. Boraston A. B. Gilbert H. J.. (2006). Differential recognition of plant cell walls by microbial xylan-specific carbohydrate-binding modules. Proc. Natl. Acad. Sci. U.S.A. 103, 4765–4770. 10.1073/pnas.0508887103 16537424 PMC1450244

[B38] McCartney L. Steele-King C. G. Jordan E. Knox J. P. (2003). Cell wall pectic (1→4)-β-D-galactan marks the acceleration of cell elongation in the *Arabidopsis* seedling root meristem. Plant J. 33, 447–454. 10.1046/j.1365-313X.2003.01640.x 12581303

[B39] McQueen-Mason S. Durachko D. M. Cosgrove D. J. (1992). 2 Endogenous proteins that induce cell-wall extension in plants. Plant Cell 4, 1425–1433. 10.1105/tpc.4.11.1425 11538167 PMC160229

[B40] Mewalal R. Mizrachi E. Mansfield S. D. Myburg A. A. (2014). Cell wall-related proteins of unknown function: missing links in plant cell wall development. Plant Cell Physiol. 55, 1031–1043. 10.1093/pcp/pcu050 24683037

[B41] Micheli F. (2001). Pectin methylesterases: cell wall enzymes with important roles in plant physiology. Trends Plant Sci. 6, 414–419. 10.1016/S1360-1385(01)02045-3 11544130

[B42] Middleton A. M. Ubeda-Tomás S. Griffiths J. Holman T. Hedden P. Thomas S. G.. (2012). Mathematical modeling elucidates the role of transcriptional feedback in gibberellin signaling. Proc. Natl. Acad. Sci. U.S.A. 109, 7571–7576. 10.1073/pnas.1113666109 22523240 PMC3358864

[B43] Miedes E. Suslov D. Vandenbussche F. Kenobi K. Ivakov A. Van Der Straeten D.. (2013). Xyloglucan endotransglucosylase/hydrolase (XTH) overexpression affects growth and cell wall mechanics in etiolated Arabidopsis hypocotyls. J. Exp. Bot. 64, 2481–2497. 10.1093/jxb/ert107 23585673

[B44] Mohler K. E. Simmons T. J. Fry S. C. (2013). Mixed-linkage glucan:xyloglucan endotransglucosylase (MXE) re-models hemicelluloses in Equisetum shoots but not in barley shoots or Equisetum callus. New Phytol. 197, 111–122. 10.1111/j.1469-8137.2012.04371.x 23078260

[B45] Moller I. Marcus S. E. Haeger A. Verhertbruggen Y. Verhoef R. Schols H. (2008). High-throughput screening of monoclonal antibodies against plant cell wall glycans by hierarchical clustering of their carbohydrate microarray binding profiles. Glycoconj. J. 25, 37–48 10.1007/s10719-007-9059-7 17629746 PMC2234451

[B46] Moller I. Sørensen I. Bernal A. J. Blaukopf C. Lee K. Øbro J.. (2007). High-throughput mapping of cell-wall polymers within and between plants using novel microarrays. Plant J. 50, 1118–1128. 10.1111/j.1365-313X.2007.03114.x 17565618

[B47] Moussaieff A. Rogachev I. Brodsky L. Malitsky S. Toal T. W. Belcher H.. (2013). High-resolution metabolic mapping of cell types in plant roots. Proc. Natl. Acad. Sci. U.S.A. 110, E1232–E1241. 10.1073/pnas.1302019110 23476065 PMC3612672

[B48] Nieuwland J. Feron R. Huisman B. A. H. Fasolino A. Hilbers C. W. Derksen J.. (2005). Lipid transfer proteins enhance cell wall extension in tobacco. Plant Cell 17, 2009–2019. 10.1105/tpc.105.032094 15937228 PMC1167548

[B49] Nishitani K. Nevins D. J. (1991). Glucuronoxylan xylanohydrolase. A unique xylanase with the requirement for appendant glucuronosyl units. J. Biol. Chem. 266, 6539–6543. 1901062

[B50] Nishitani K. Vissenberg K. (2007). Roles of the XTH protein family in the expanding cell, in The Expanding Cell. Plant Cell Monographs, Vol. 5, eds Verbelen J.-P. Vissenberg K. (Berlin; Heidelberg; New York: Springer), 89–116.

[B51] Okamoto-Nakazato A. Takahashi K. Katoh-Semba R. Katou K. (2001). Distribution of yieldin a regulatory protein of the cell wall yield threshold in etiolated cowpea seedlings. Plant Cell Physiol. 42, 952–958. 10.1093/pcp/pce121 11577189

[B52] Passardi F. Penel C. Dunand C. (2004). Performing the paradoxical: how plant peroxidases modify the cell wall. Trends Plant Sci. 9, 534–540. 10.1016/j.tplants.2004.09.002 15501178

[B53] Passardi F. Tognolli M. De Meyer M. Penel C. Dunand C. (2006). Two cell wall associated peroxidases from Arabidopsis influence root elongation. Planta 223, 965–974. 10.1007/s00425-005-0153-4 16284776

[B54] Peret B. Li G. Zhao J. Band L. R. Voss U. Postaire O.. (2012). Auxin regulates aquaporin function to facilitate lateral root emergence. Nat. Cell Biol. 14, 991–998. 10.1038/ncb2573 22983115

[B55] Pérez-Rodrłguez P. Riaño-Pachón D. M. Corrêa L. G. Rensing S. A. Kersten B. Mueller-Roeber B. (2010). PlnTFDB: updated content and new features of the plant transcription factor database. Nucl. Acids Res. 38, D822–D827. 10.1093/nar/gkp805 19858103 PMC2808933

[B56] Perrot-Rechenmann C. (2010). Cellular responses to auxin: division versus expansion. Cold Spring Harb. Perspect. Biol. 2:a001446. 10.1101/cshperspect.a001446 20452959 PMC2857164

[B57] Ray P. M. Green P. B. Cleland R. (1972). Role of turgor in plant cell growth. Nature 239, 163–164 10.1038/239163a0

[B59] Rose J. K. Braam J. Fry S. C. Nishitani K. (2002). The XTH family of enzymes involved in xyloglucan endotransglucosylation and endohydrolysis: current perspectives and a new unifying nomenclature. Plant Cell Physiol. 43, 1421–1435. 10.1093/pcp/pcf171 12514239

[B60] Scheller H. V. Ulvskov P. (2010). Hemicelluloses. Annu. Rev. Plant Biol. 61, 263–289. 10.1146/annurev-arplant-042809-112315 20192742

[B61] Schopfer P. (2006). Biomechanics of plant growth. Am. J. Bot. 93, 1415–1425. 10.3732/ajb.93.10.1415 21642088

[B62] Shin I. Park S. Lee M-R. (2005). Carbohydrate microarrays: an advanced technology for functional studies of glycans. Chem. Eur. J. 11, 2894–2901. 10.1002/chem.200401030 15635679

[B63] Smyth G. K. (2005). Limma: linear models for microarray data in Bioinformatics and Computational Biology Solutions Using R and Bioconductor, eds Gentleman R. Carey V. Dudoit S. Irizarry R. Huber W. (New York, NY: Springer), 397–420 10.1007/0-387-29362-0_23

[B64] Somerville C. Bauer S. Brininstool G. Facette M. Hamann T. Milne J.. (2004). Toward a systems approach to understanding plant cell walls. Science 2306, 2206–2211. 10.1126/science.1102765 15618507

[B65] Sørensen I. Willats W. G. T. (2011). Screening and characterization of plant cell walls using carbohydrate microarrays, in Methods in Molecular Biology, Vol. 715, ed Popper Z. (John Walker) (New York, NY: Humana Press), 115–121. 10.1007/978-1-61779-008-9_8 21222080

[B66] Talboys P. J. Zhang H. M. Knox J. P. (2011). ABA signaling modulates the detection of the LM6 arabinan cell wall epitope at the surface of *Arabidopsis thaliana* seedling root apices. New Phytol. 190, 618–626. 10.1111/j.1469-8137.2010.03625.x 21275992

[B67] Ubeda-Thomás S. Federici F. Casimiro I. Beemster G. T. S. Bhalerao R. Swarup R.. (2009). Gibberellin signaling in the endodermis controls Arabidopsis root meristem size. Curr. Biol. 19, 1194–1199. 10.1016/j.cub.2009.06.023 19576770

[B68] Van Sandt V. Suslov D. Verbelen J.-P. Vissenberg K. (2007). Xyloglucan endotransglucosylase activity loosens a plant cell wall. Ann. Bot. 100, 1467–1473. 10.1093/aob/mcm248 17916584 PMC2759230

[B69] Verbelen J.-P. De Cnodder T. Le J. Vissenberg K. Baluška F. (2006). Root apex of *Arabidopsis thaliana* consists of four distinct zones of growth activities: meristematic zone, transition zone, fast elongation zone, and growth terminating zone. Plant Signal. Behav. 1, 296–304. 10.4161/psb.1.6.3511 19517000 PMC2634244

[B70] Vissenberg K. Martinez-Vilchez I. M. Verbelen J.-P. Miller J. G. Fry S. C. (2000). *In vivo* colocalisation of xyloglucan endotransglycosylase activity and its donor substrate in the elongation zone of Arabidopsis roots. Plant Cell 12, 1229–1237 10.1105/tpc.12.7.1229 10899986 PMC149061

[B71] Vissenberg K. Oyama M. Osato Y. Yokoyama R. Verbelen J. P. Nishitani K. (2005). Differential expression of AtXTH17, AtXTH18, AtXTH19 and AtXTH20 genes in *Arabidopsis* roots. Physiological roles in specification in cell wall construction. Plant Cell Physiol. 46, 192–200. 10.1093/pcp/pci013 15659443

[B72] Willats W. G. McCartney L. Steele-King C. G. Marcus S. E. Mort A. Huisman M.. (2008). A xylogalacturonan epitope is specifically associated with plant cell detachment. Planta 218, 673–681. 10.1007/s00425-003-1147-8 14618325

