# Supplementary material for: Multi-omics analysis identifies genes mediating the extension of cell walls in the Arabidopsis thaliana root elongation zone
Source: Front Cell Dev Biol. 2015 Feb 20;3:10. doi: 10.3389/fcell.2015.00010 (PMC4335395; doi:10.3389/fcell.2015.00010)
Supplement: Supplementary file 1 [file Table1.PDF]

Supplementary Table 1. Transcriptomic data for the five zones of the *A. thaliana* root. A gene was considered to be expressed if its expression was greater than 100 and differentially expressed if a t-test between two zones was significant at a q-value of 0.05.

Total	Zone 1	Zone 2	Zone 3	Zone 4	Zone 5
Expressed	7741 (36.3%)	7539 (35.3%)	7801 (36.6%)	7854 (36.8%)	8044 (37.7%)
	Z1→Z2	Z2→Z3	Z3→Z4	Z4→Z5	Z5→Z1
DE	1632(up) 1939(dn)	1343 (up) 1750 (dn)	155/369	155/184	1322/2627
TF: 1574 annotated genes on the array					
Expressed	349 (22.2%)	366 (23.3%)	408 (25.9%)	401 (25.5%)	392 (24.9%)
DE	46/83	99/42	5/19	8/9	73/82
Cell Wall: 1043 annotated genes on the array					
Expressed	367 (35.2%)	426 (40.8%)	415 (39.8%)	407 (39.0%)	402 (38.5%)
DE	147/40	65/110	15/33	15/23	63/86
ROS: 100 annotated genes on the array					
Expressed	21 (21.0%)	36 (36.0%)	52 (52.0%)	48 (48.0%)	48 (48.0%)
DE	5/2	14/5	1/5	1/5	5.4
AQP: 30 annotated genes on the array					
Expressed	8 (26.7%)	17 (56.7%)	19 (63.3%)	17 (56.7%)	17 (56.7%)
DE	7/0	7/2	0/4	0/3	6/1
Hormone: 999 annotated genes on the array					
Expressed	383 (38.3%)	423 (42.3%)	476 (47.6%)	473 (47.3%)	492 (49.2%)
DE	85 (up) 78 (dn)	94 (up) 82 (dn)	10 (up) 20 (dn)	16 (up) 8 (dn)	92 (up) 115 (dn)
