# Supplementary material for: Multi-omics analysis identifies genes mediating the extension of cell walls in the Arabidopsis thaliana root elongation zone
Source: Front Cell Dev Biol. 2015 Feb 20;3:10. doi: 10.3389/fcell.2015.00010 (PMC4335395; doi:10.3389/fcell.2015.00010)

in situ immunolocalisation of Cell Wall epitope antibodies

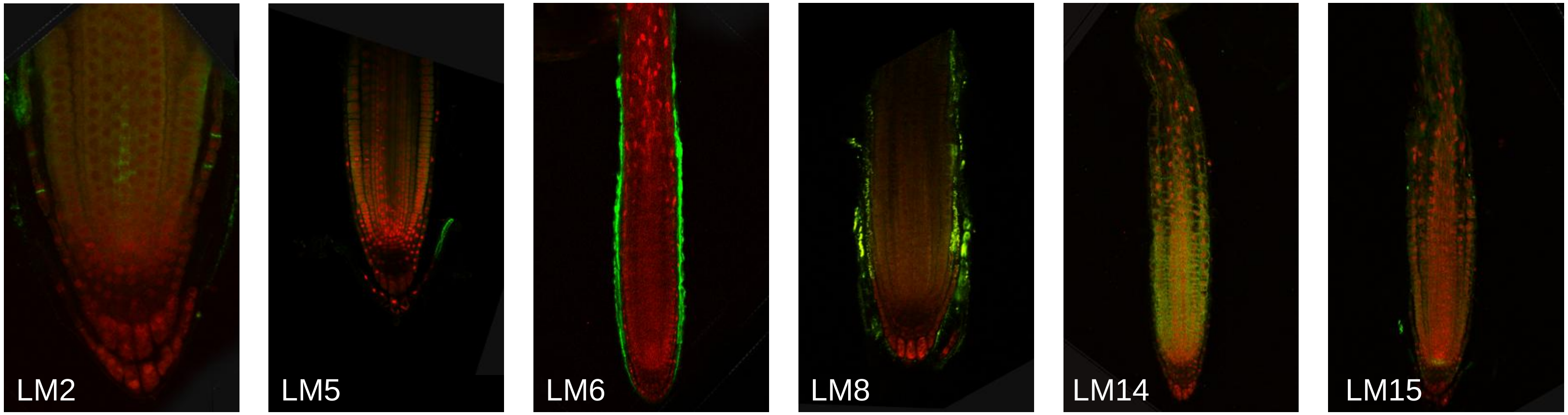

No signal could be detected for LM 1, 7, 9, 10, 11, 12 &13

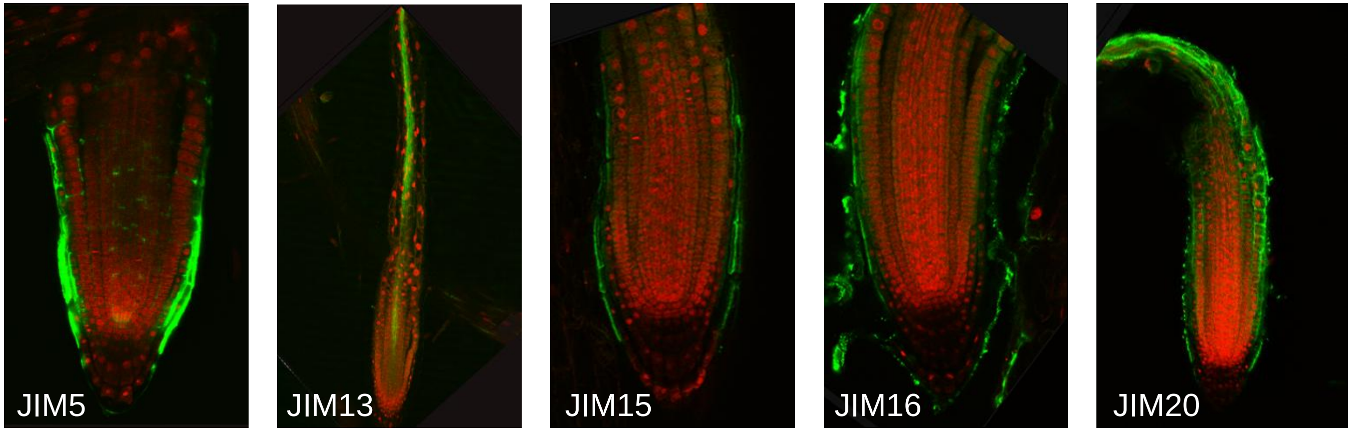

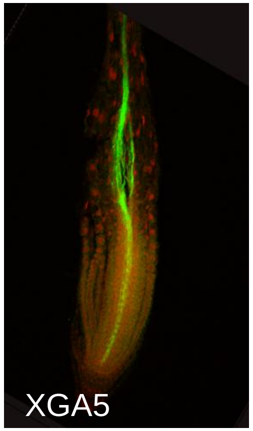

No signal could be detected for JIM 4, 7, 8, 12, 19

No signal for XGA4

in situ immunolocalisation of Cell Wall epitope antibodies

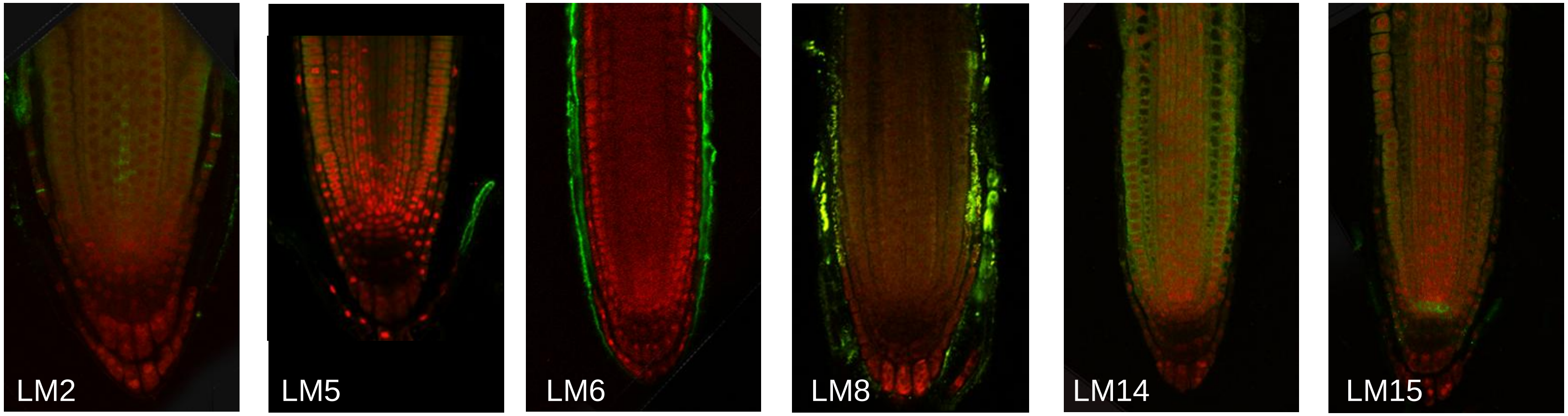

No signal could be detected for LM 1, 7, 9, 10, 11, 12 &13

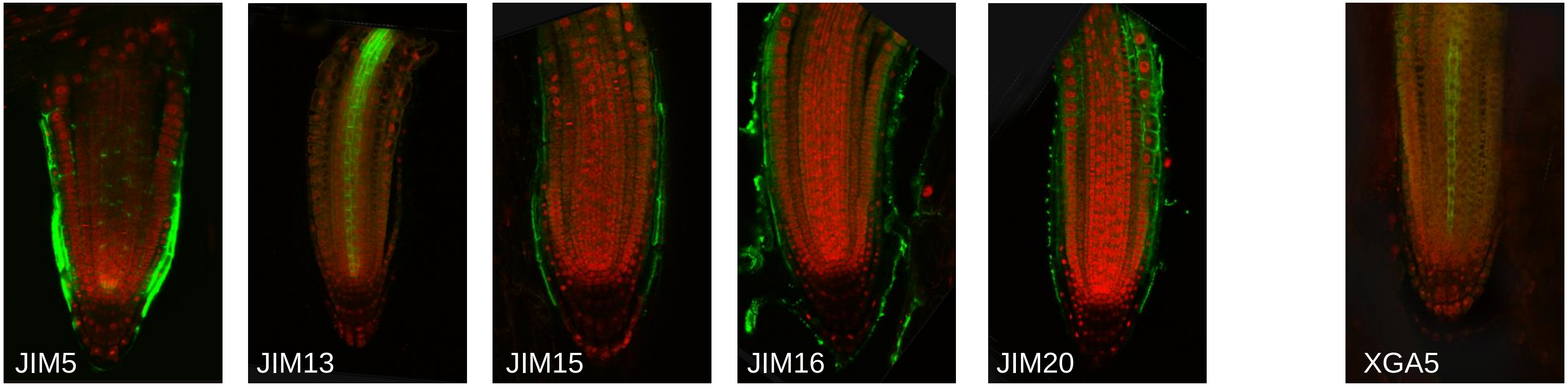

No signal could be detected for JIM 4, 7, 8, 12, 19

No signal for XGA4

in situ immunolocalisation of Cell Wall epitope antibodies

Summary: CCRC-M series

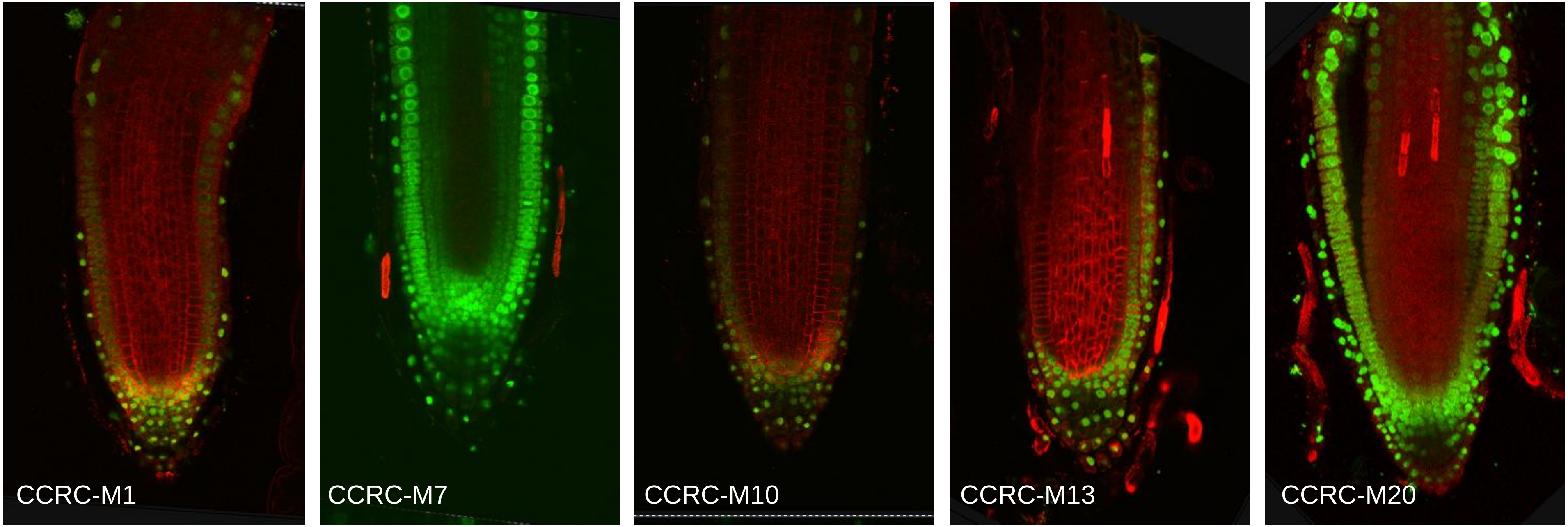

No signal could be detected for CCRC-M2, 8 and 70

Anti CCRC-M 1:100
Anti Mouse AF543 1:200

JIM5

MONOCLONAL ANTIBODY to HOMOGALACTURONAN

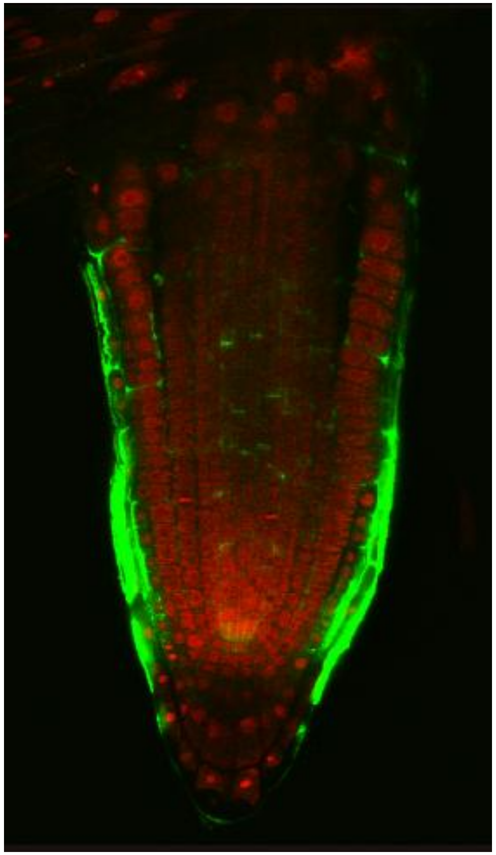

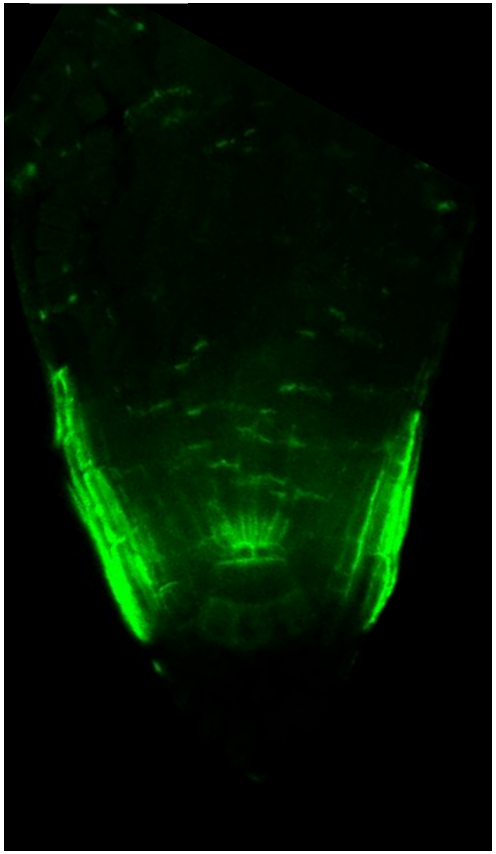

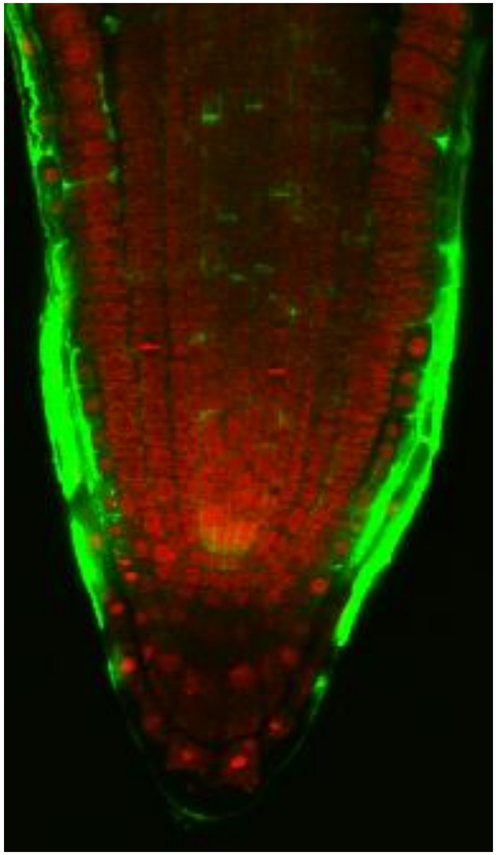

Anti JIM5 1:100

Anti Rat AF488 1:200

Scale bar 100 µm

JIM13

Monoclonal Antibody To Arabinogalactan Protein

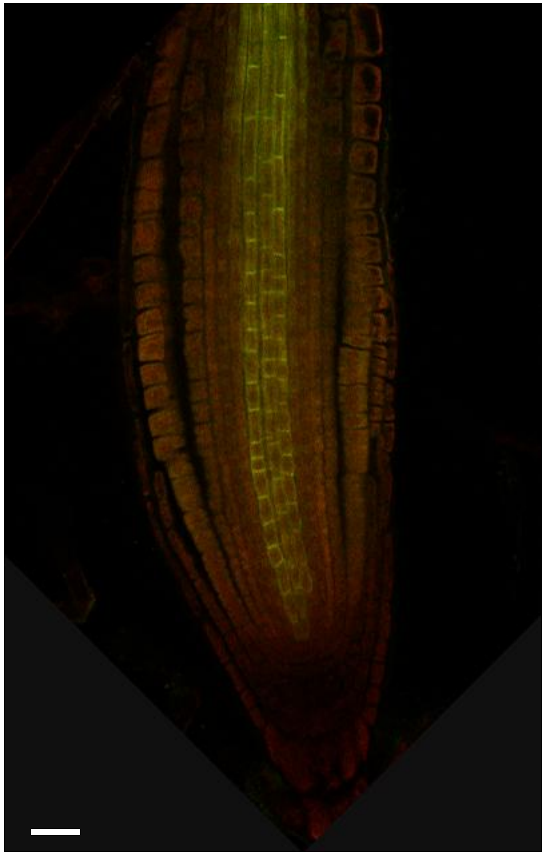

Anti JIM13 1:100

Anti Rat AF488 1:200

Scale bar 100 µm

JIM13

Monoclonal Antibody To Arabinogalactan Protein

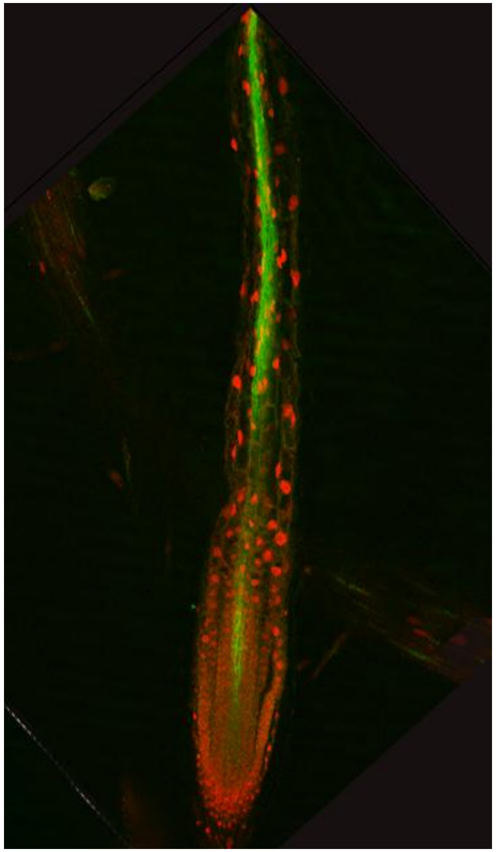

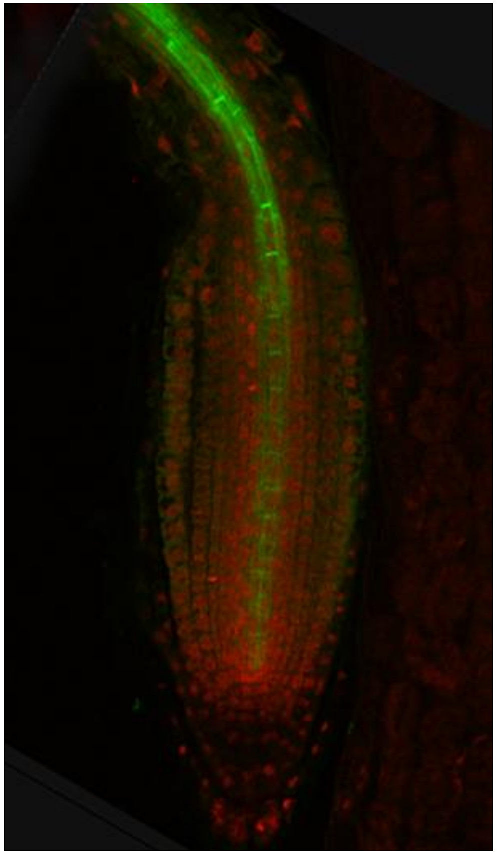

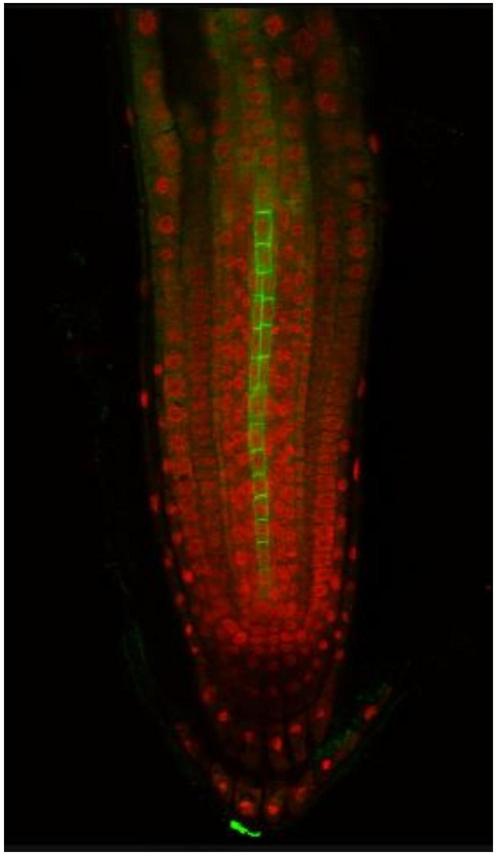

Anti JIM13 1:100
Anti Rat AF488 1:200

Scale bar 100 µm

JIM13

Monoclonal Antibody To Arabinogalactan Protein

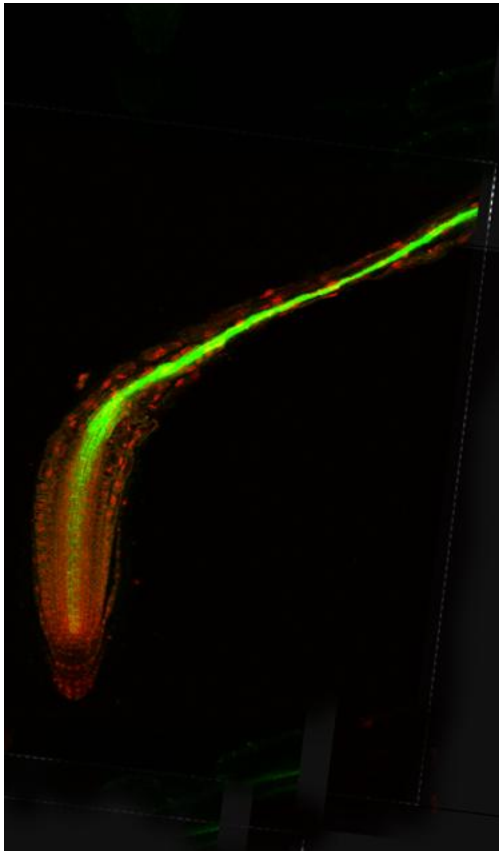
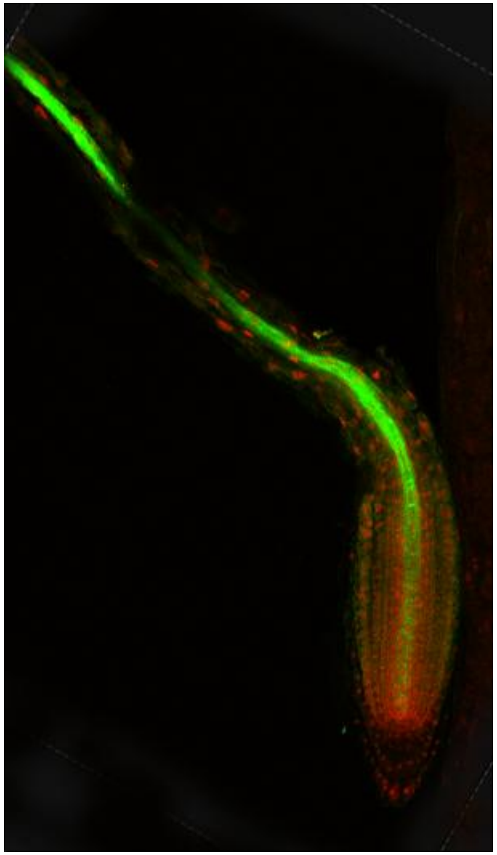
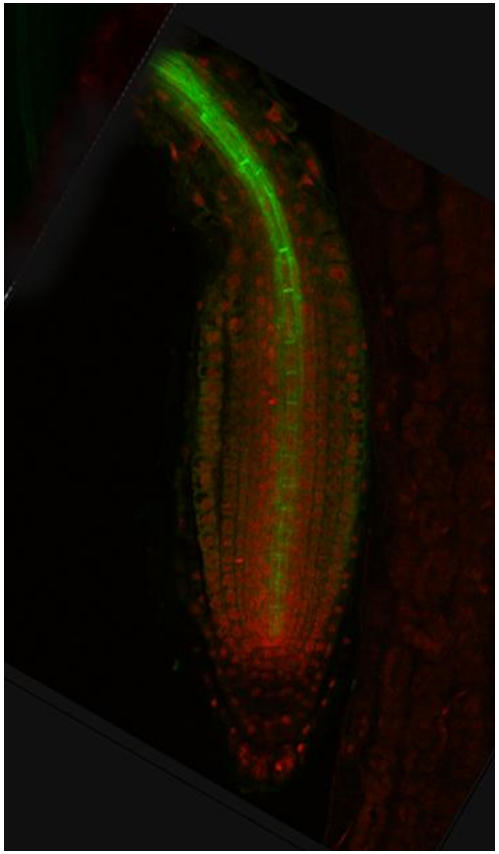

Anti JIM13 1:100
Anti Rat AF488 1:200

Scale bar 100 μm

JIM13

Monoclonal Antibody To Arabinogalactan Protein

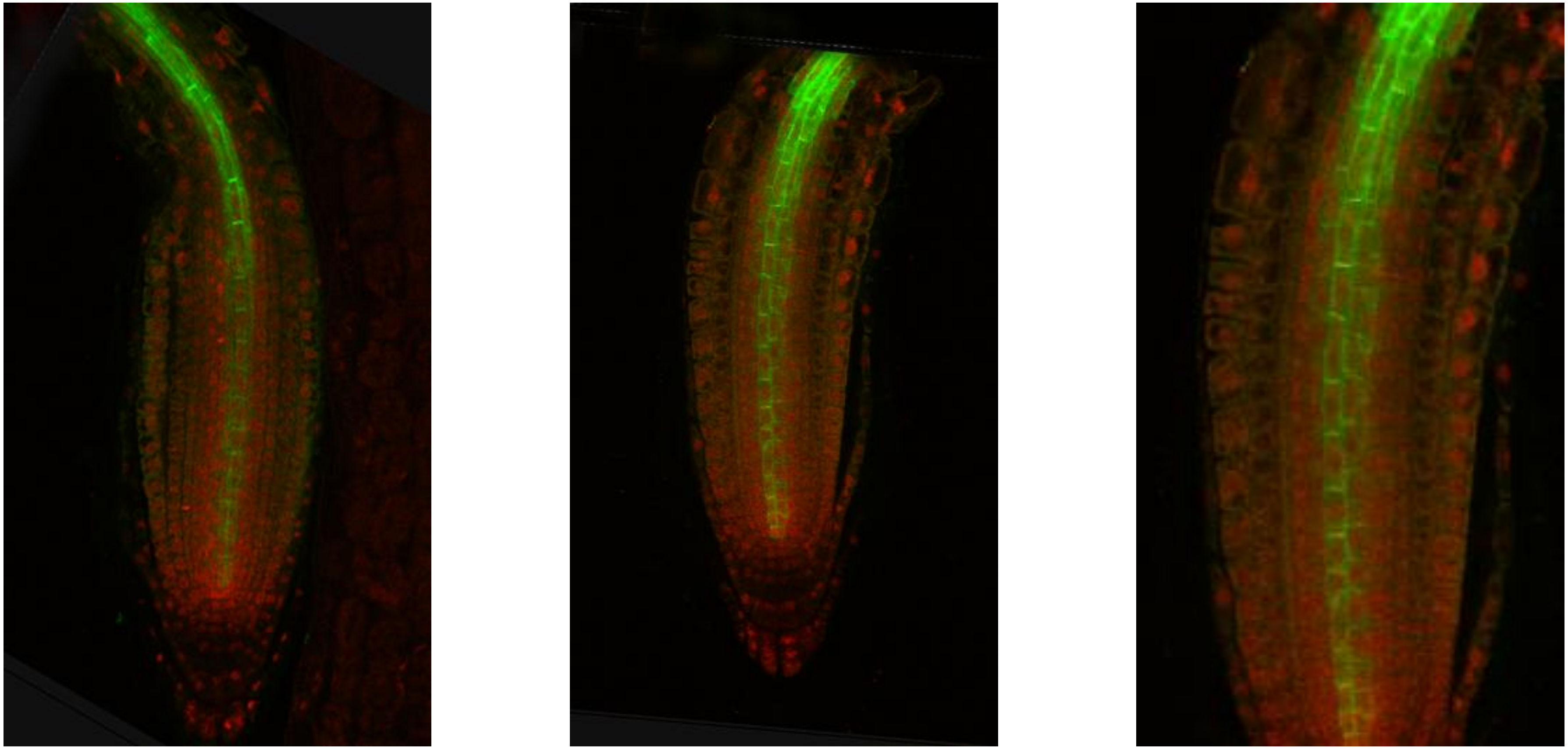

Anti JIM13 1:100
Anti Rat AF488 1:200

Scale bar 100 µm

JIM15

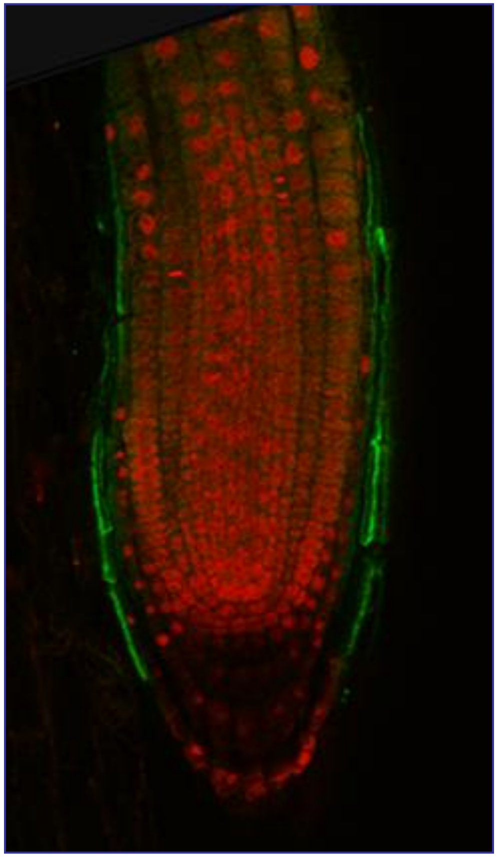

Anti JIM15 1:100
Anti Rat AF488 1:200

Scale bar 100 µm

JIM16

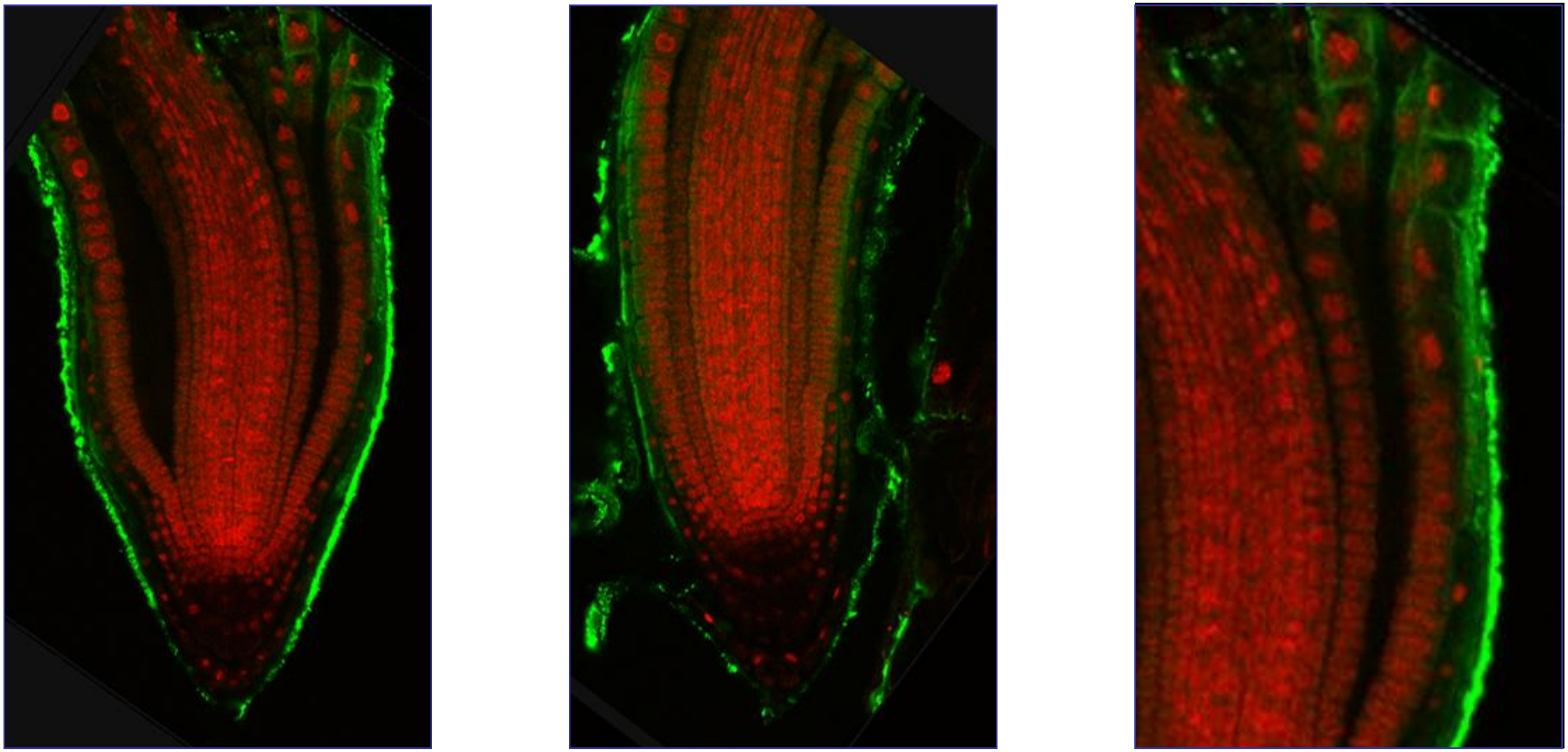

Anti JIM16 1:100
Anti Rat AF488 1:200

Scale bar 100 µm

JIM20

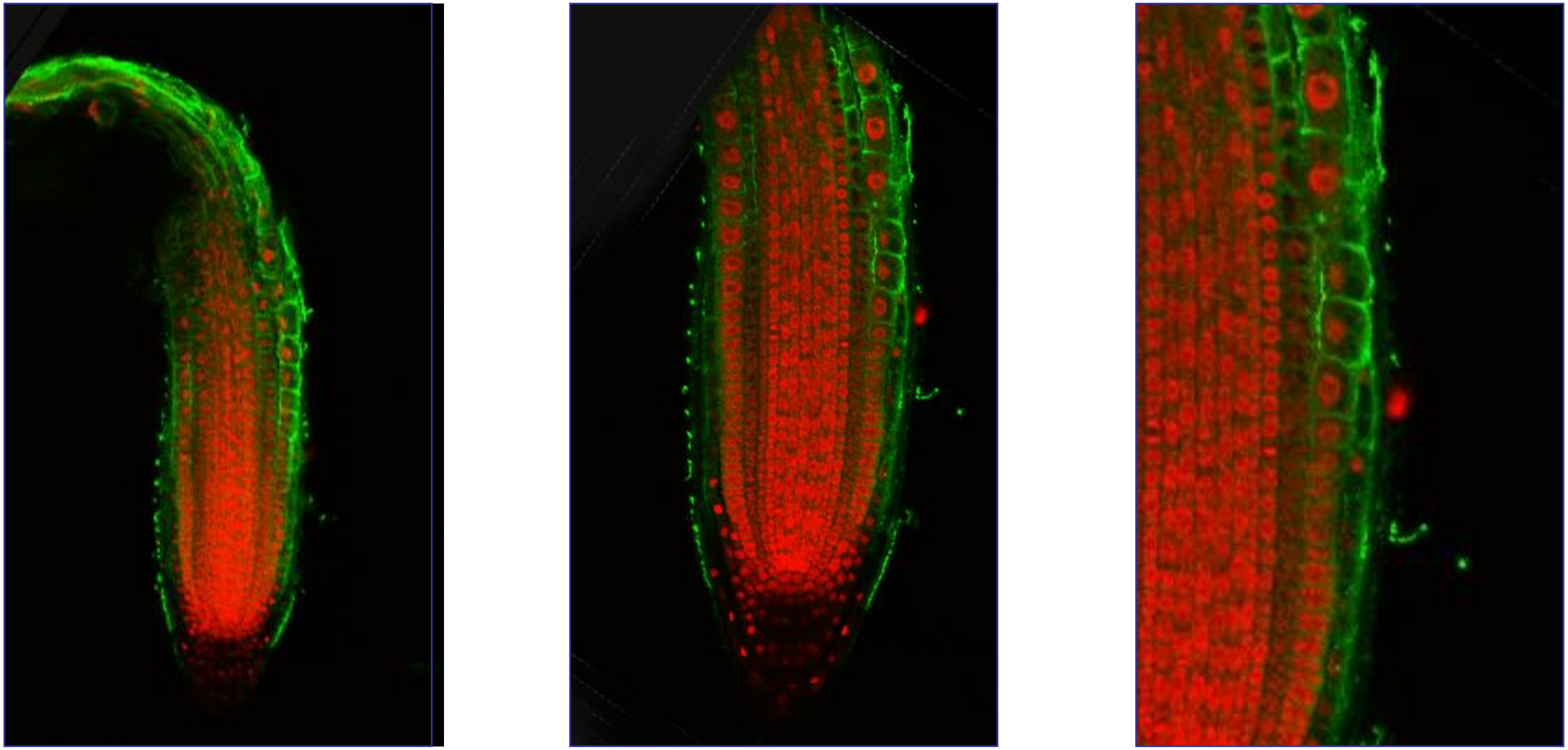

Anti JIM20 1:100
Anti Rat AF488 1:200

Scale bar 100 µm

LM2

ARABINOGALACTAN-PROTEIN

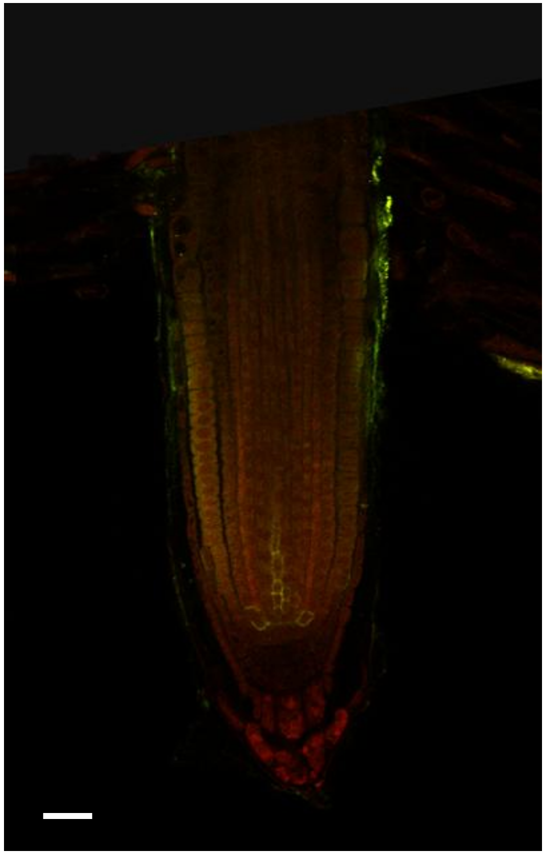

Anti LM2 1:100
Anti Rat AF488 1:200

Scale bar 50 μm

LM2

MONOCLONAL ANTIBODY to ARABINOGALACTAN PROTEIN

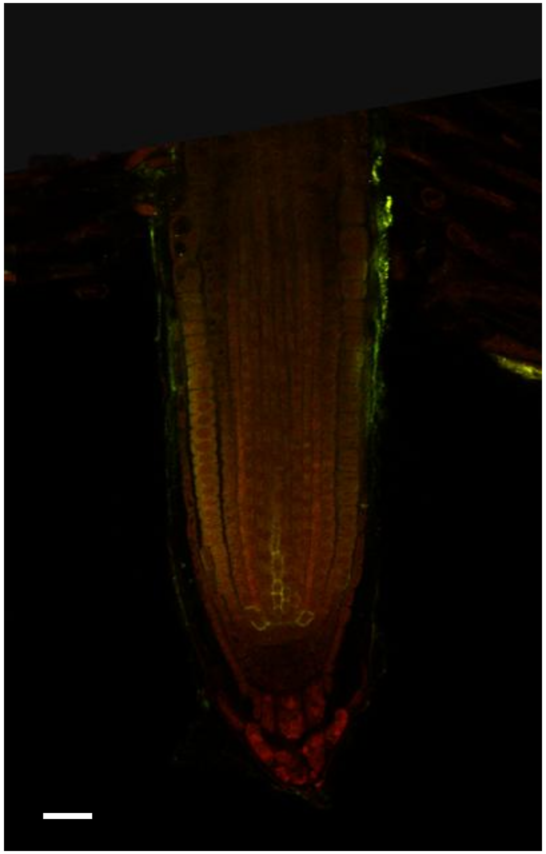

Anti LM2 1:100
Anti Rat AF488 1:200

Scale bar 50 μm

LM2

MONOCLONAL ANTIBODY to ARABINOGALACTAN PROTEIN

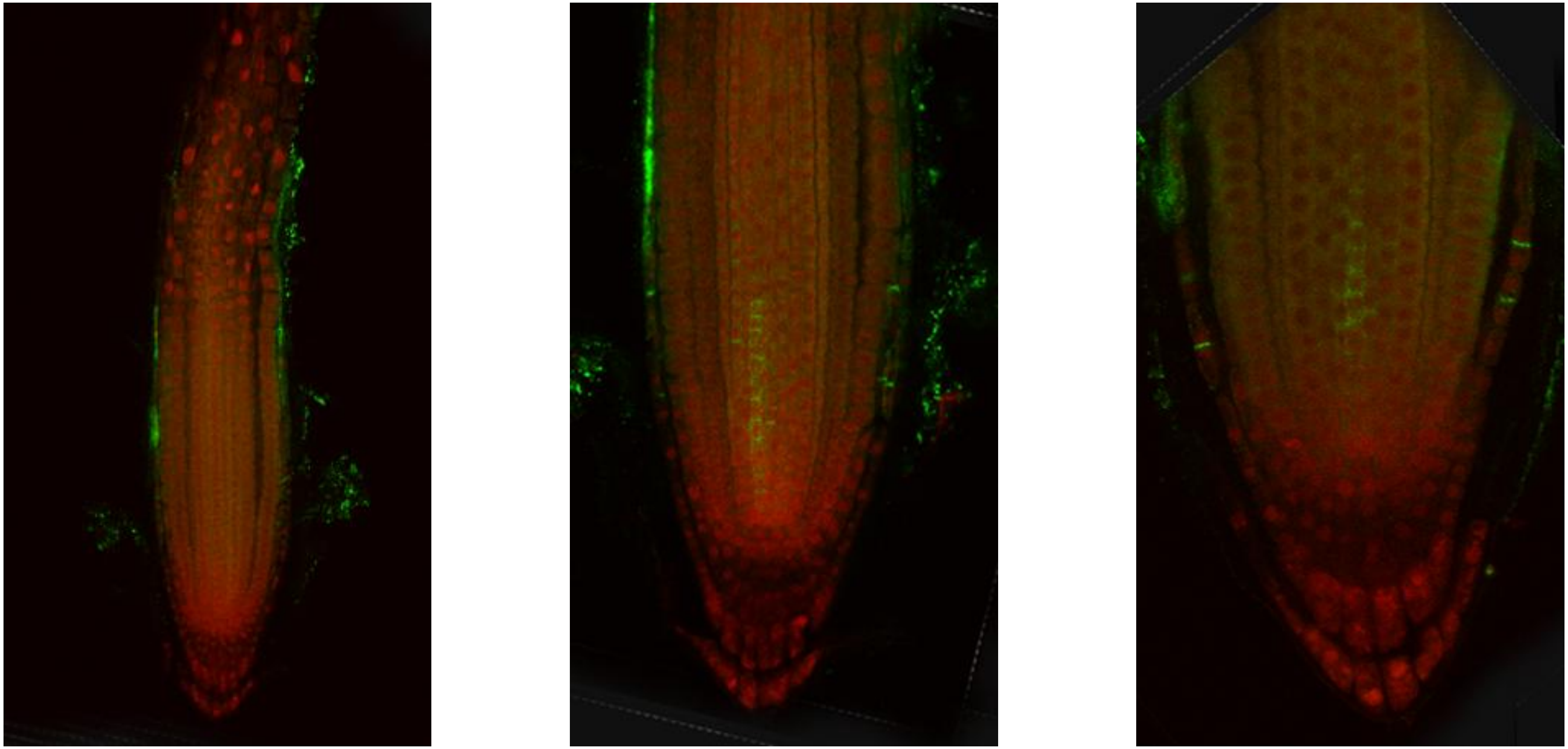

Anti LM2 1:100
Anti Rat AF488 1:200

LM2

MONOCLONAL ANTIBODY to ARABINOGALACTAN PROTEIN

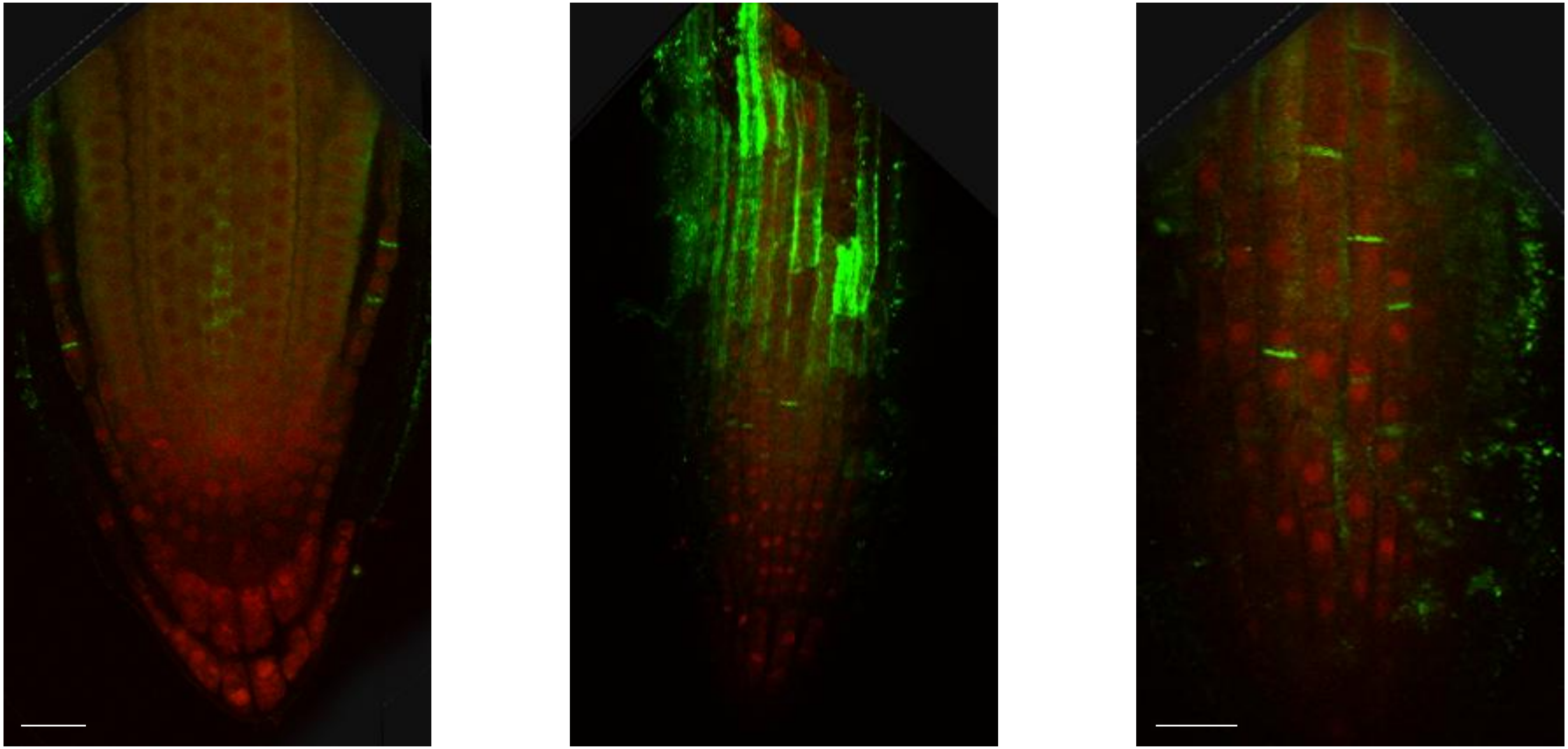

Anti LM2 1:100
Anti Rat AF488 1:200

Scale bar 20 µm

LM2

MONOCLONAL ANTIBODY to ARABINOGALACTAN PROTEIN

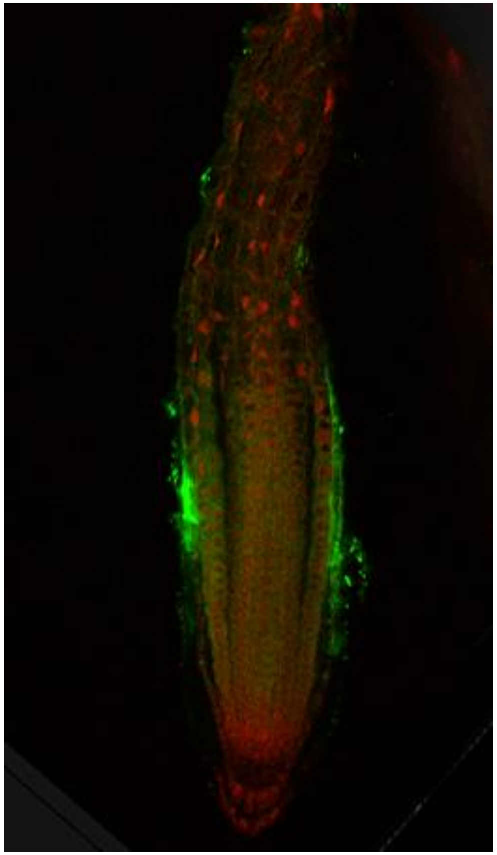

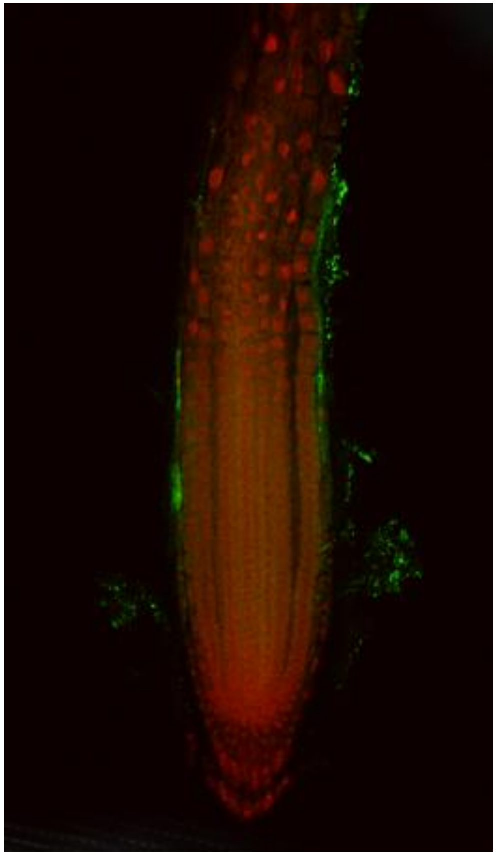

Anti LM2 1:100

Anti Rat AF488 1:200

Scale bar 50 μm

LM2

MONOCLONAL ANTIBODY to ARABINOGALACTAN PROTEIN

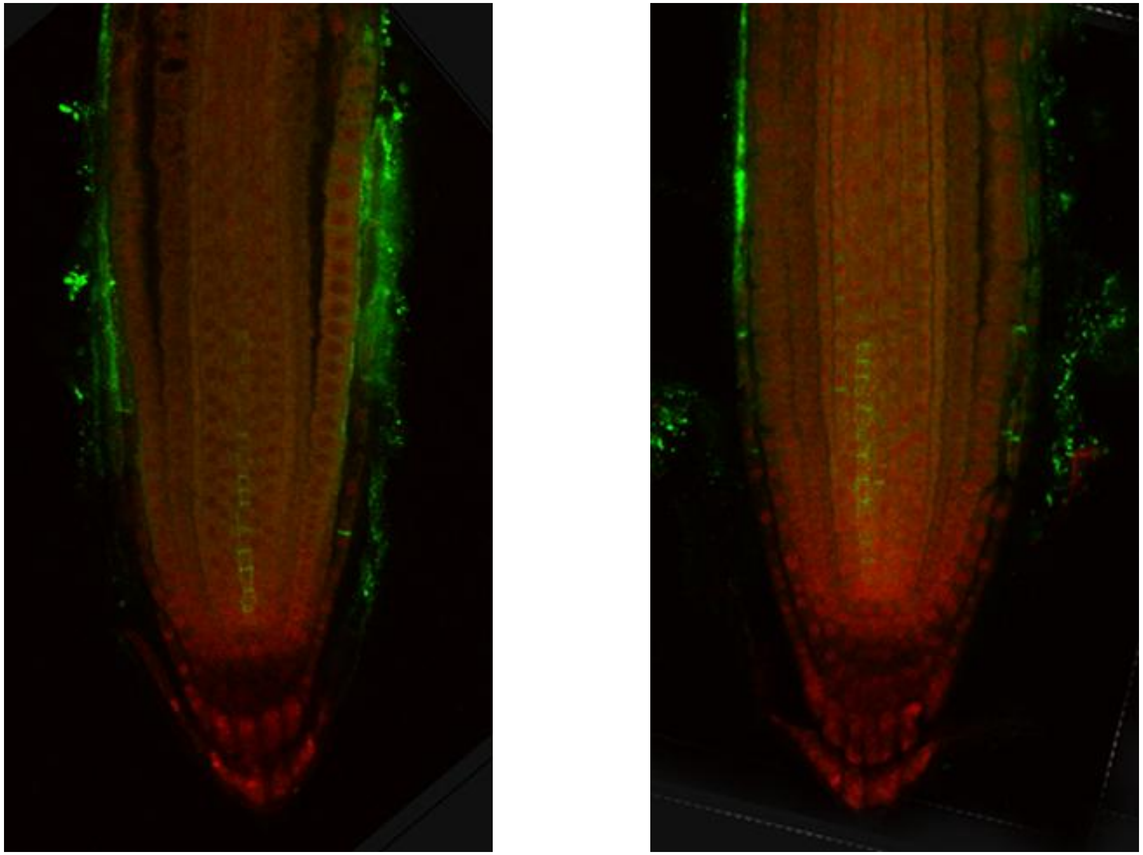

Anti LM2 1:100
Anti Rat AF488 1:200

Scale bar 50 μm

LM5

MONOCLONAL ANTIBODY to (1-4)-ß-D-GALACTAN

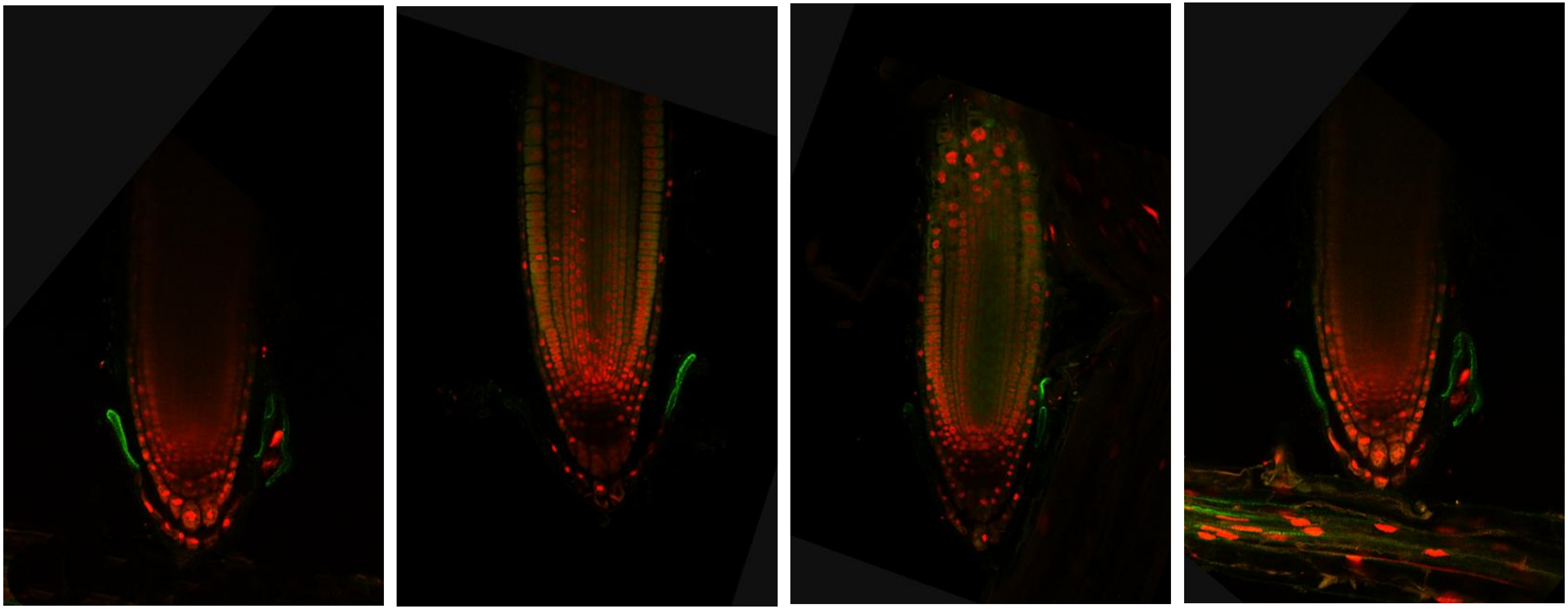

Anti LM5 1:100
Anti Rat AF488 1:200

Scale bar 50 μm

LM6

MONOCLONAL ANTIBODY to (1-5)-α-L-ARABINAN

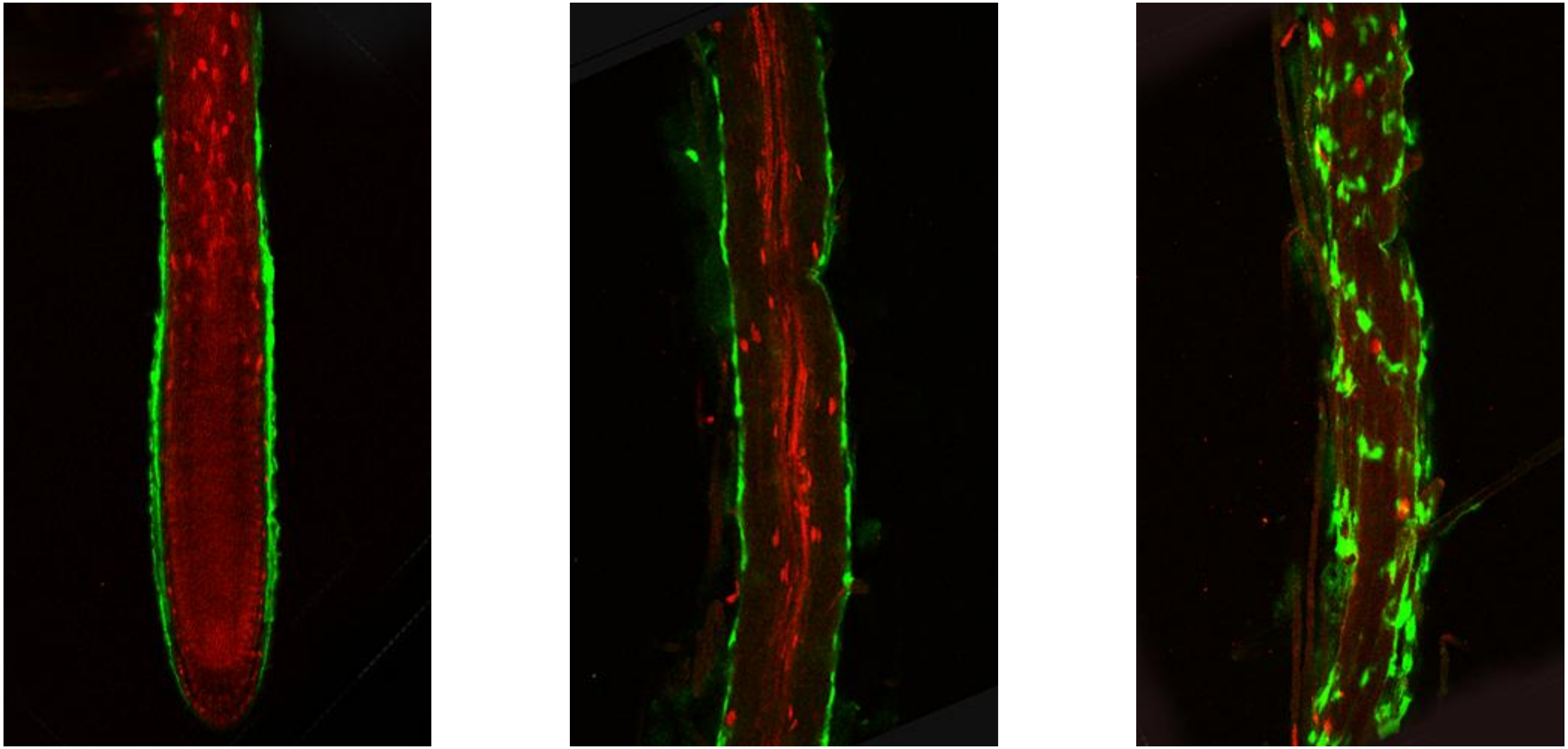

Anti LM6 1:100
Anti Rat AF488 1:200

Scale bar 50 μm

LM6

MONOCLONAL ANTIBODY to (1-5)-α-L-ARABINAN

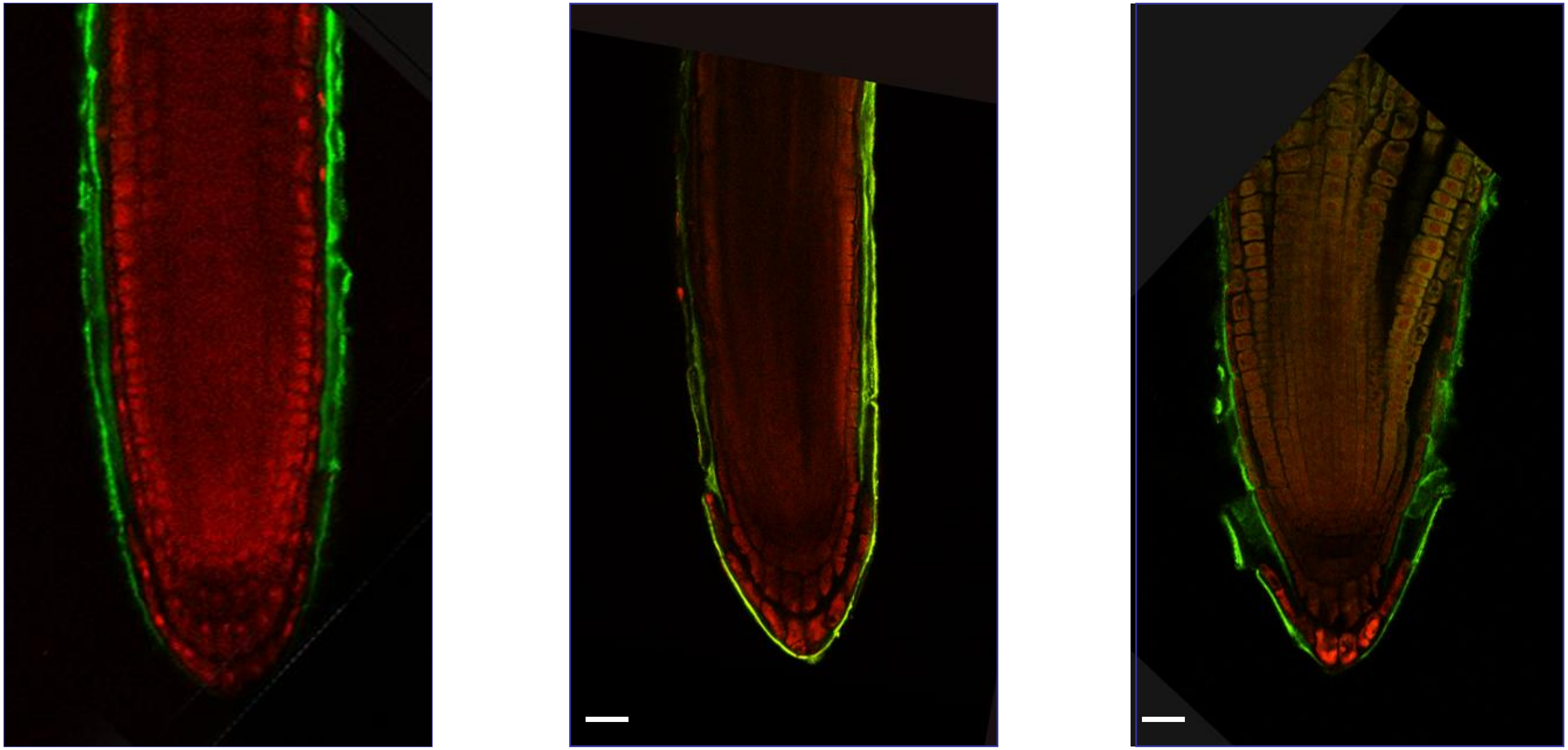

Anti LM6 1:100
Anti Rat AF488 1:200

Scale bar 50 μm

LM8

MONOCLONAL ANTIBODY to XYLOGALACTURONAN

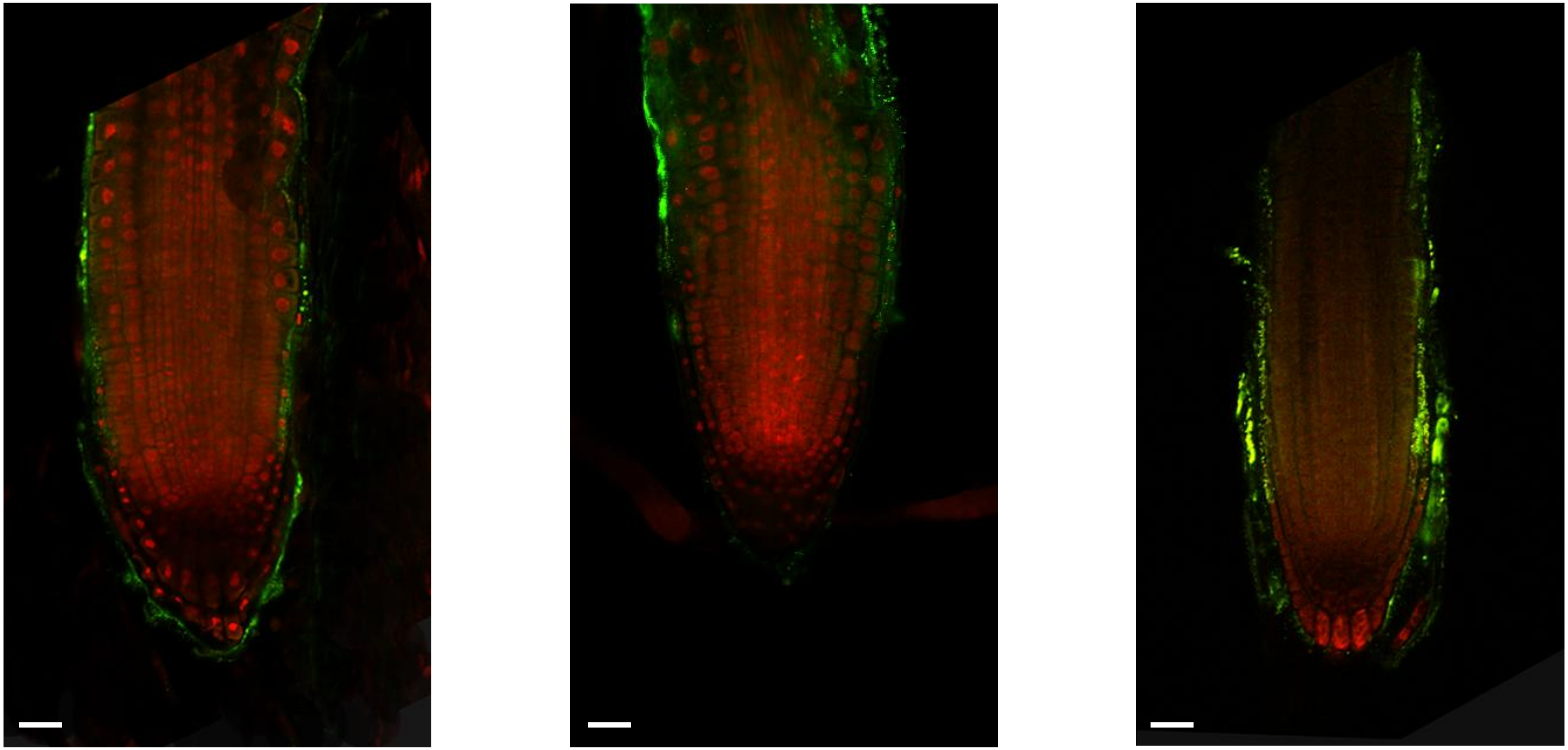

Anti LM8 1:100
Anti Rat AF488 1:200

Scale bar 50 µm

LM14

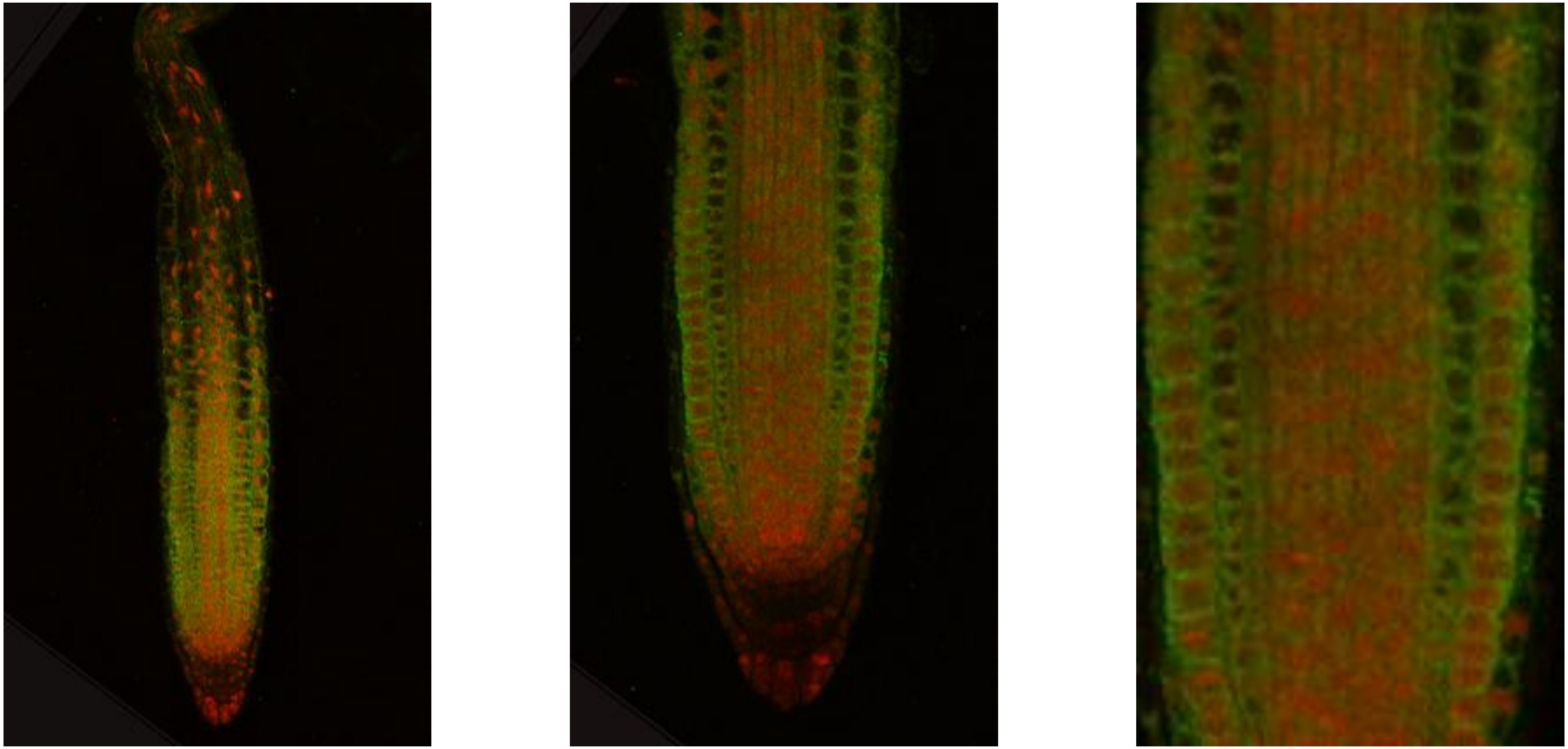

Anti LM14 1:100
Anti Rat AF488 1:200

Scale bar 100 µm

LM15

MONOCLONAL ANTIBODY to XYLOGLUCAN

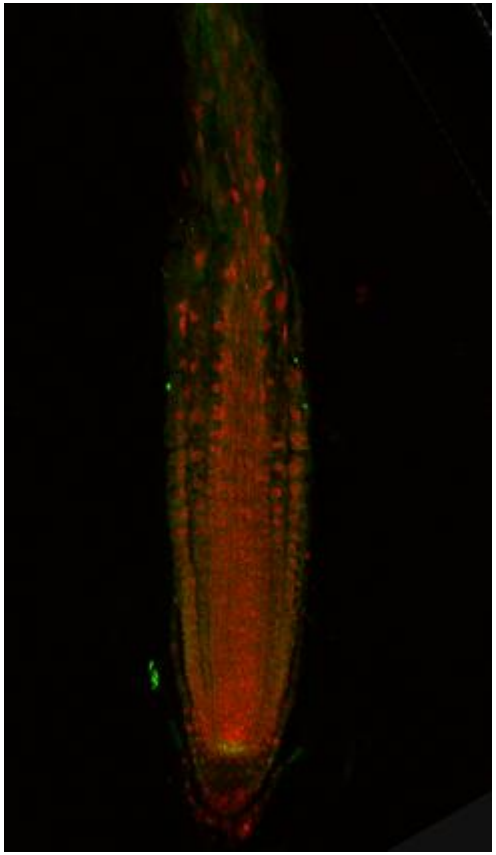

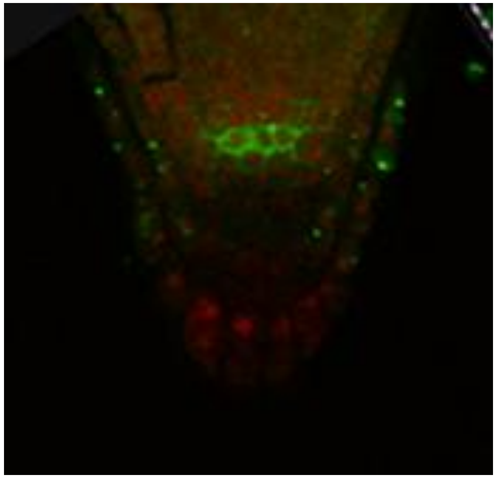

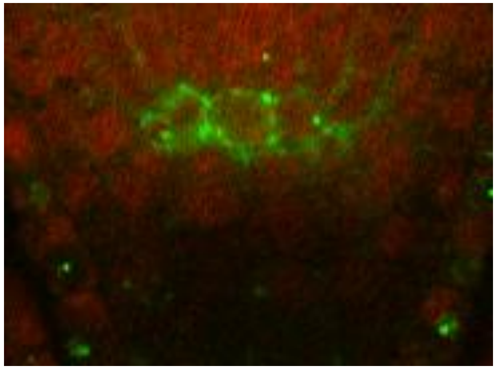

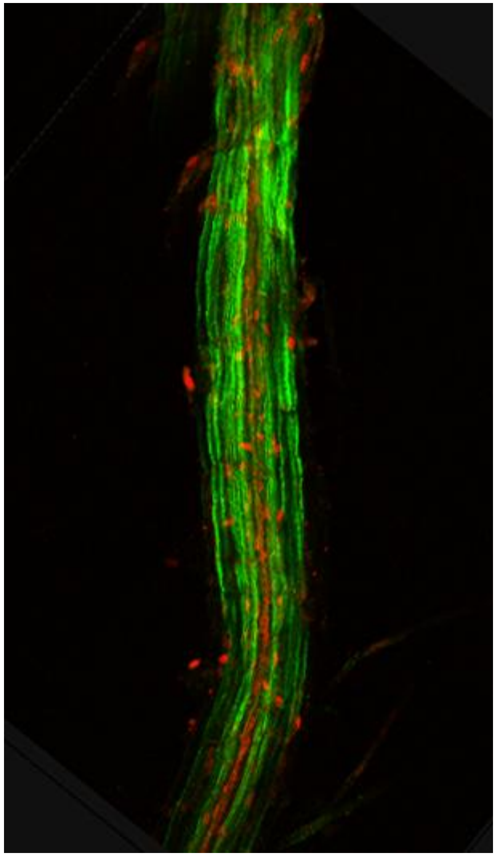

Anti LM15 1:100
Anti Rat AF488 1:200

Scale bar 100 µm

LM15

MONOCLONAL ANTIBODY to XYLOGLUCAN

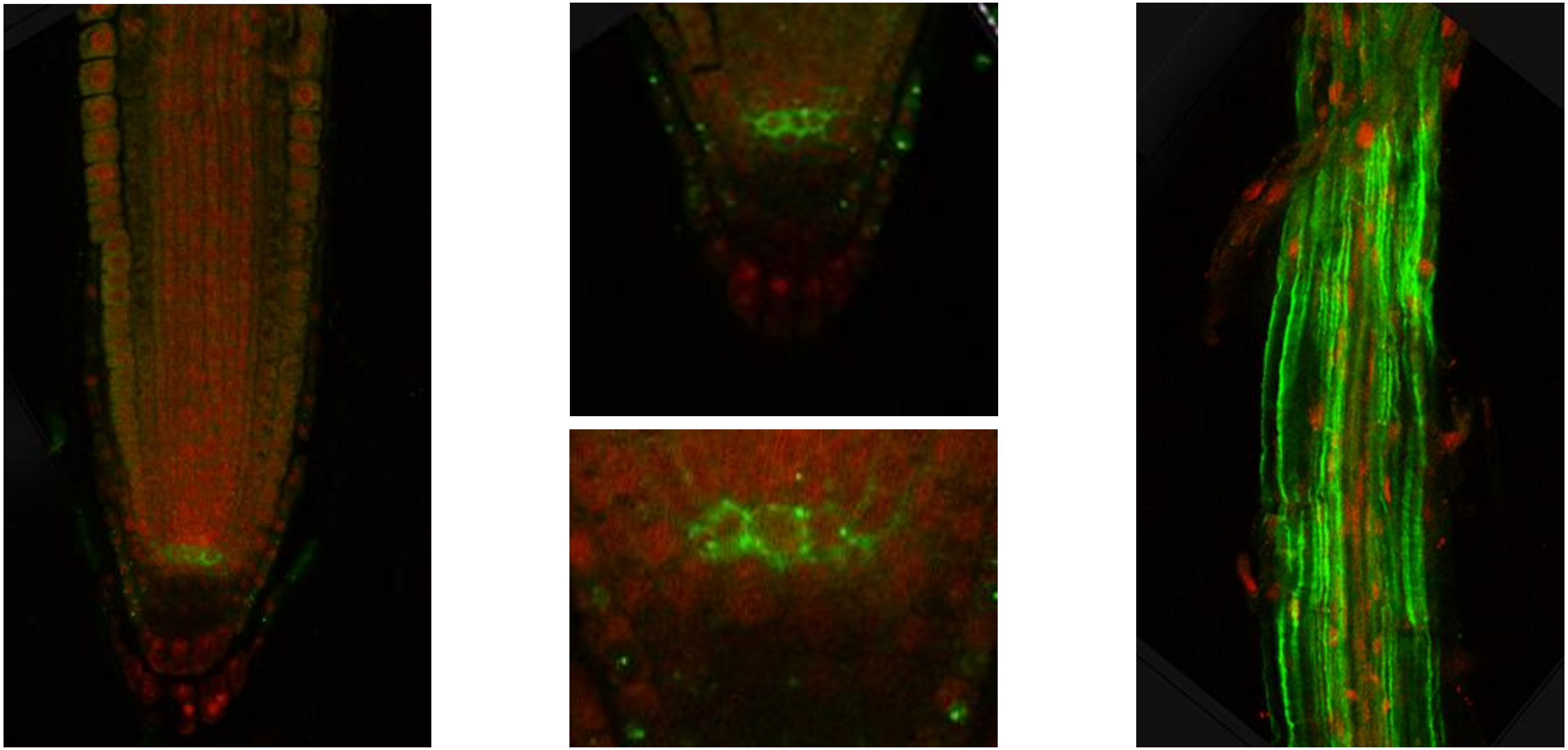

Anti LM15 1:100
Anti Rat AF488 1:200

Scale bar 100 µm

LM15

MONOCLONAL ANTIBODY to XYLOGLUCAN

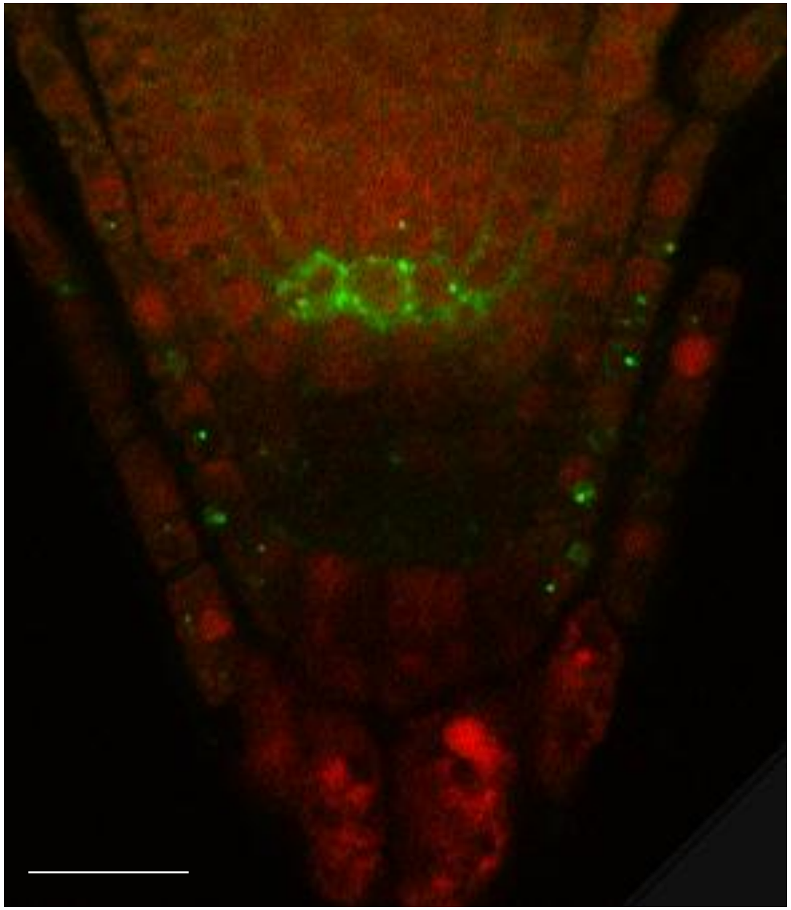

Anti LM15 1:100
Anti Rat AF488 1:200

Scale bar 20 μm

XGA5

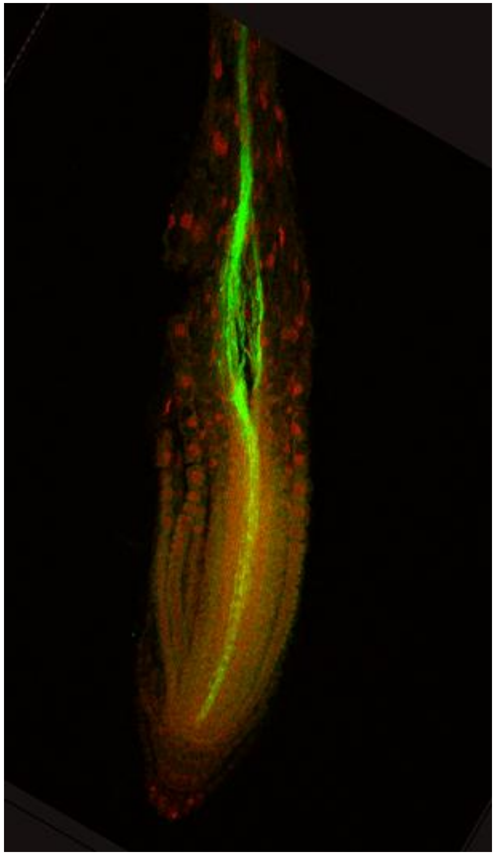

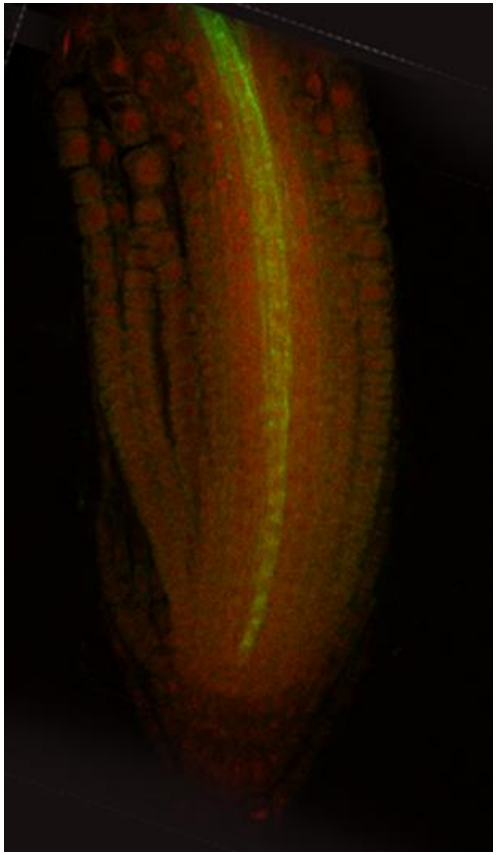

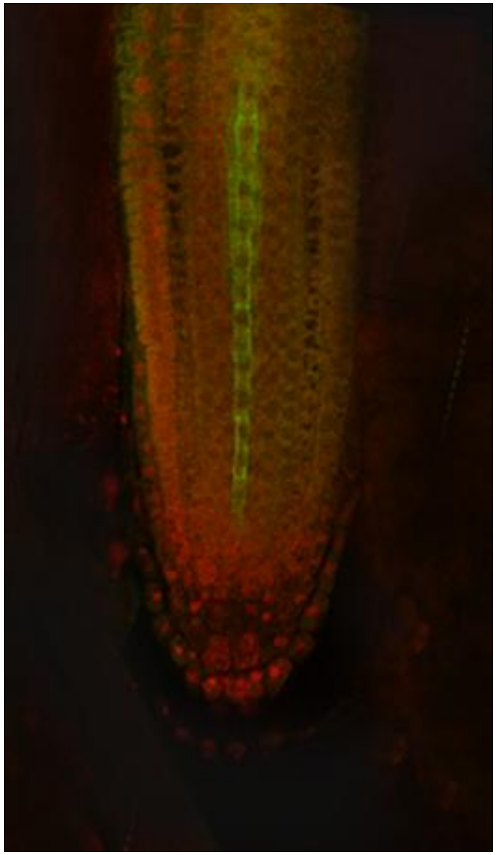

Anti XGA5 1:100
Anti Rat AF488 1:200

Scale bar 100 µm

CCRC-M1

recognizes α-L-fucosylated xyloglucan

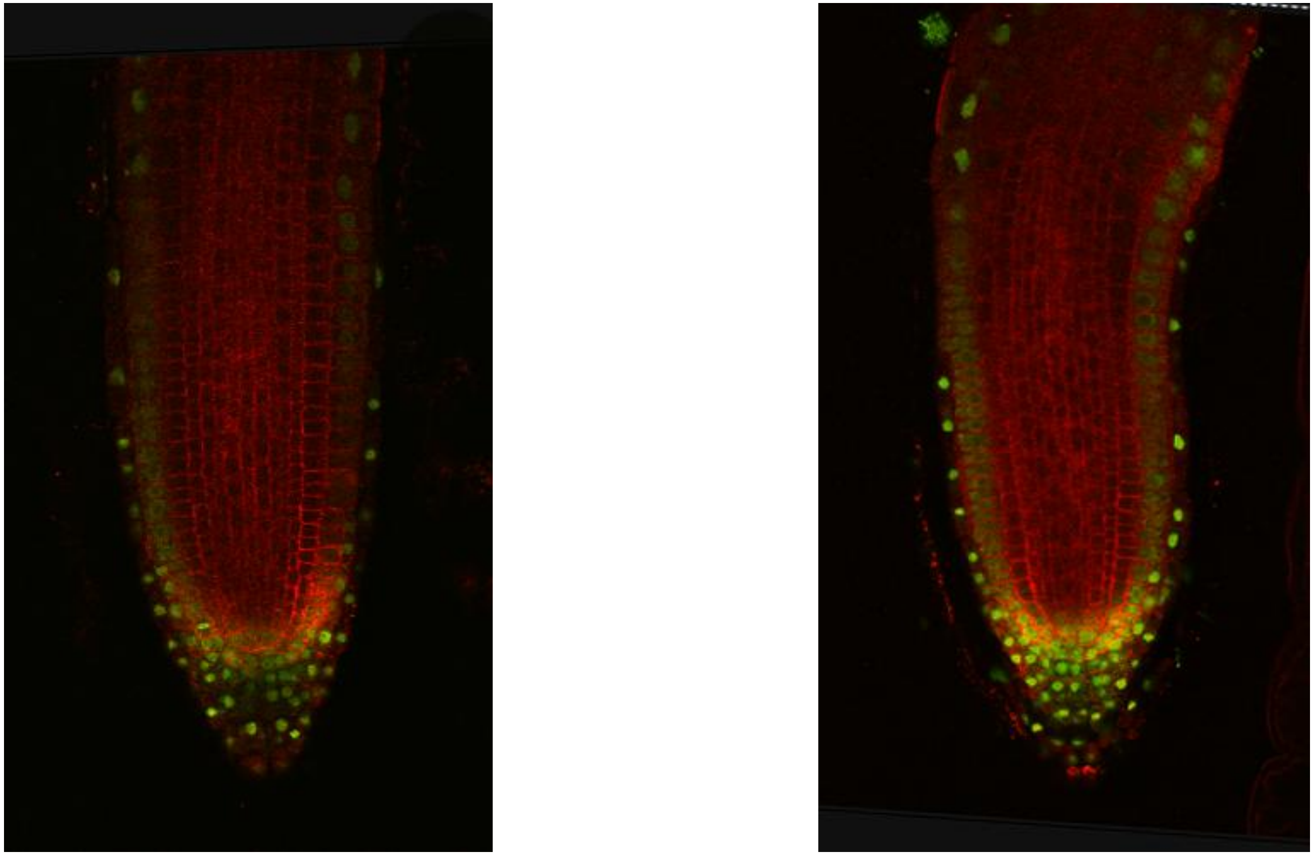

CCRC-M series

CCRC-M1 - recognizes α-L-fucosylated xyloglucan (**CS**)

CCRC-M2 - epitope not determined (**CS**)

CCRC-M7 - recognizes 6-linked β-D-galactose oligomers that contain arabinose (**CS**)
CCRC-M8 - epitope not determined (**CS**)
CCRC-M10 - epitope not determined (**CS**)
CCRC-M13 - epitope not determined (**CS**)
CCRC-M22 - epitope not determined (**CS**)
CCRC-M30 - epitope not determined (**CS**)
CCRC-M31 - epitope not determined (**CS**)
CCRC-M32 - epitope not determined (**CS**)
CCRC-M34 - epitope not determined (**CS**)
CCRC-M36 - epitope not determined (**CS**)
CCRC-M37 - epitope not determined (**CS**)
CCRC-M38 - epitope not determined (**CS**)
CCRC-M70 - epitope not determined (**CS**)

CCRC-M7

recognizes 6-linked β-D-galactose oligomers that contain arabinose

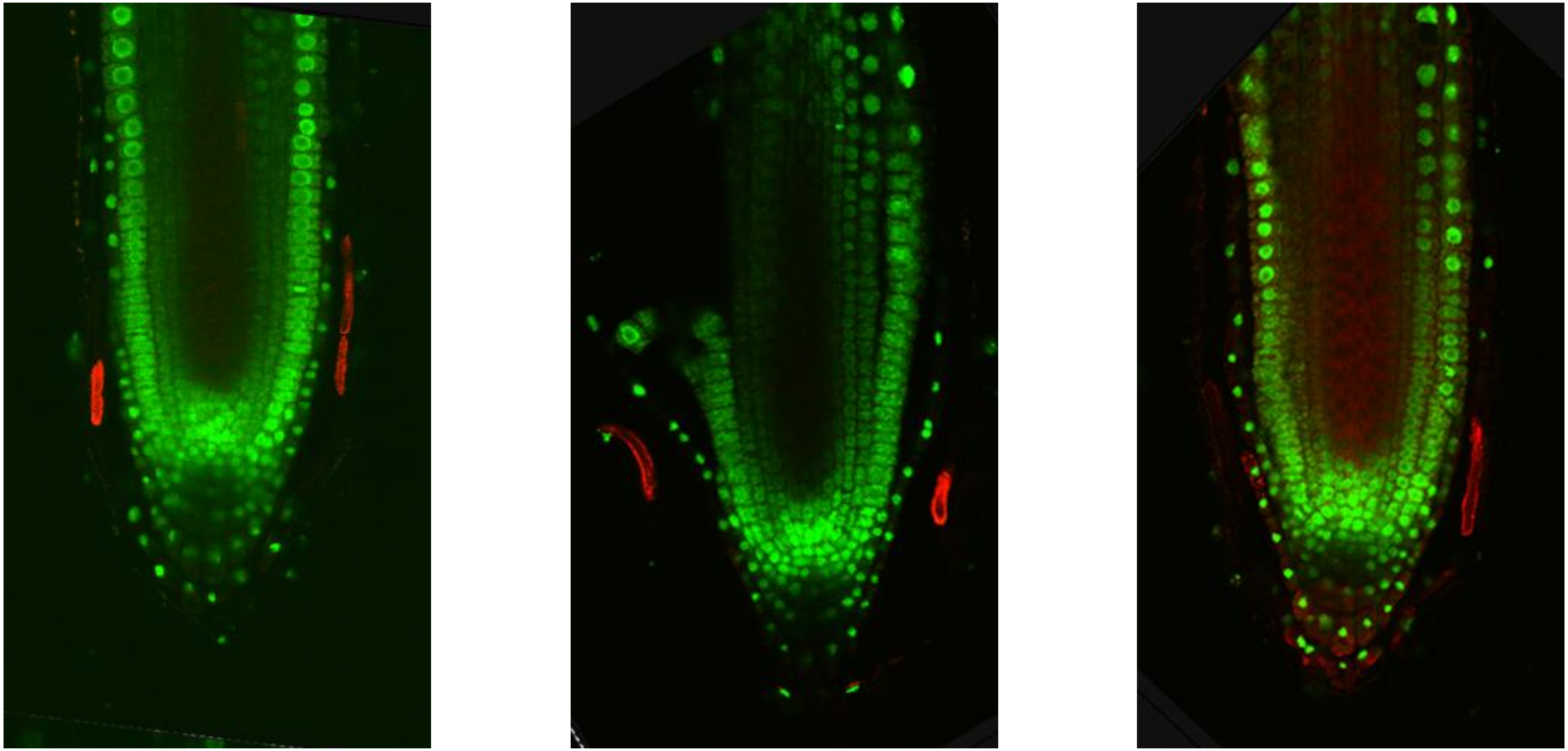

[CCRC-M7](#)

recognizes 6-linked β-D-galactose oligomers that contain arabinose

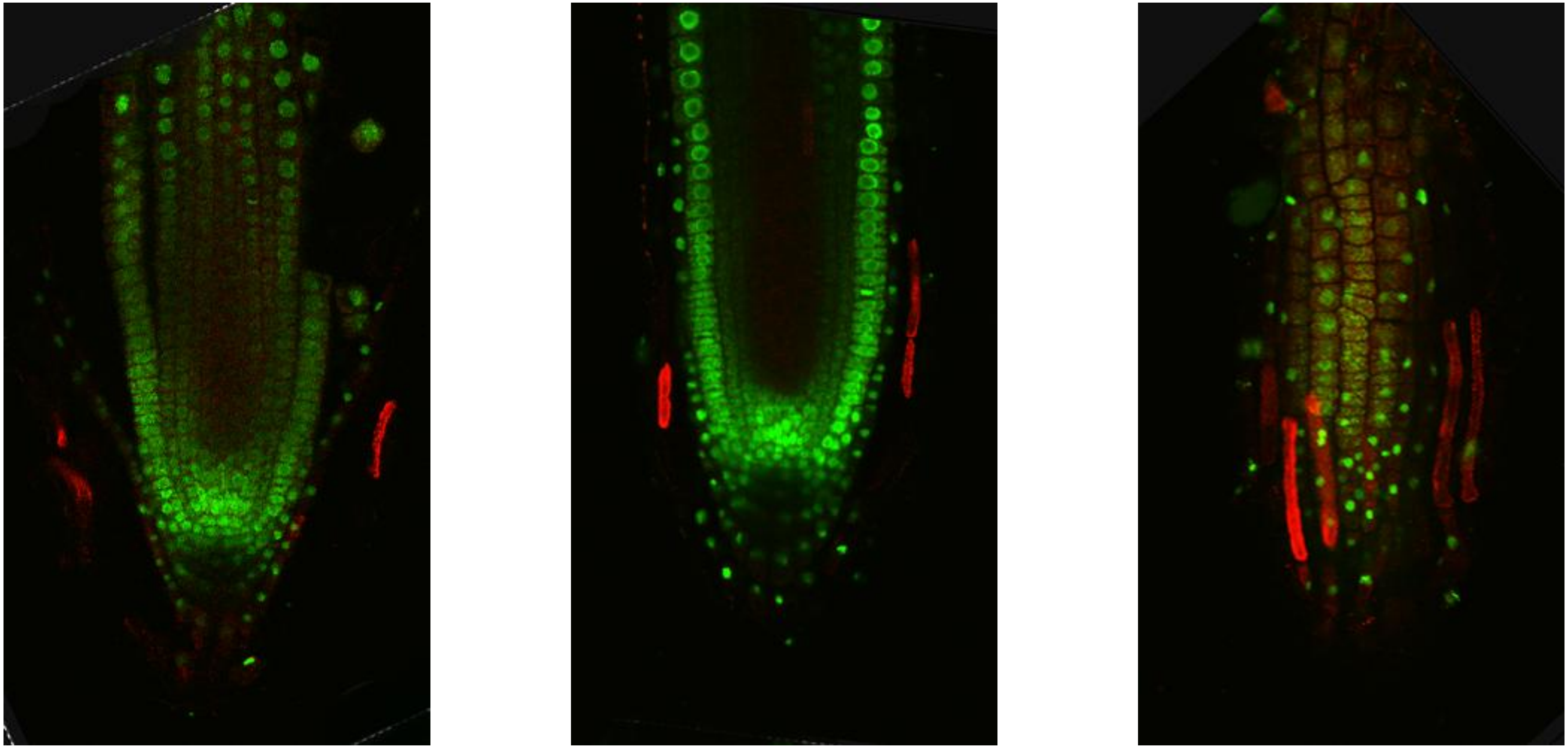

CCRC-M11

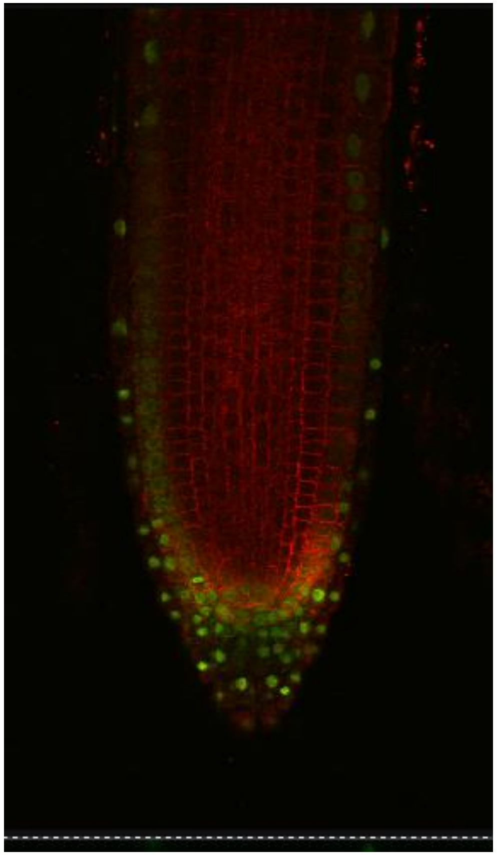

CCRC-M13

epitope not determined

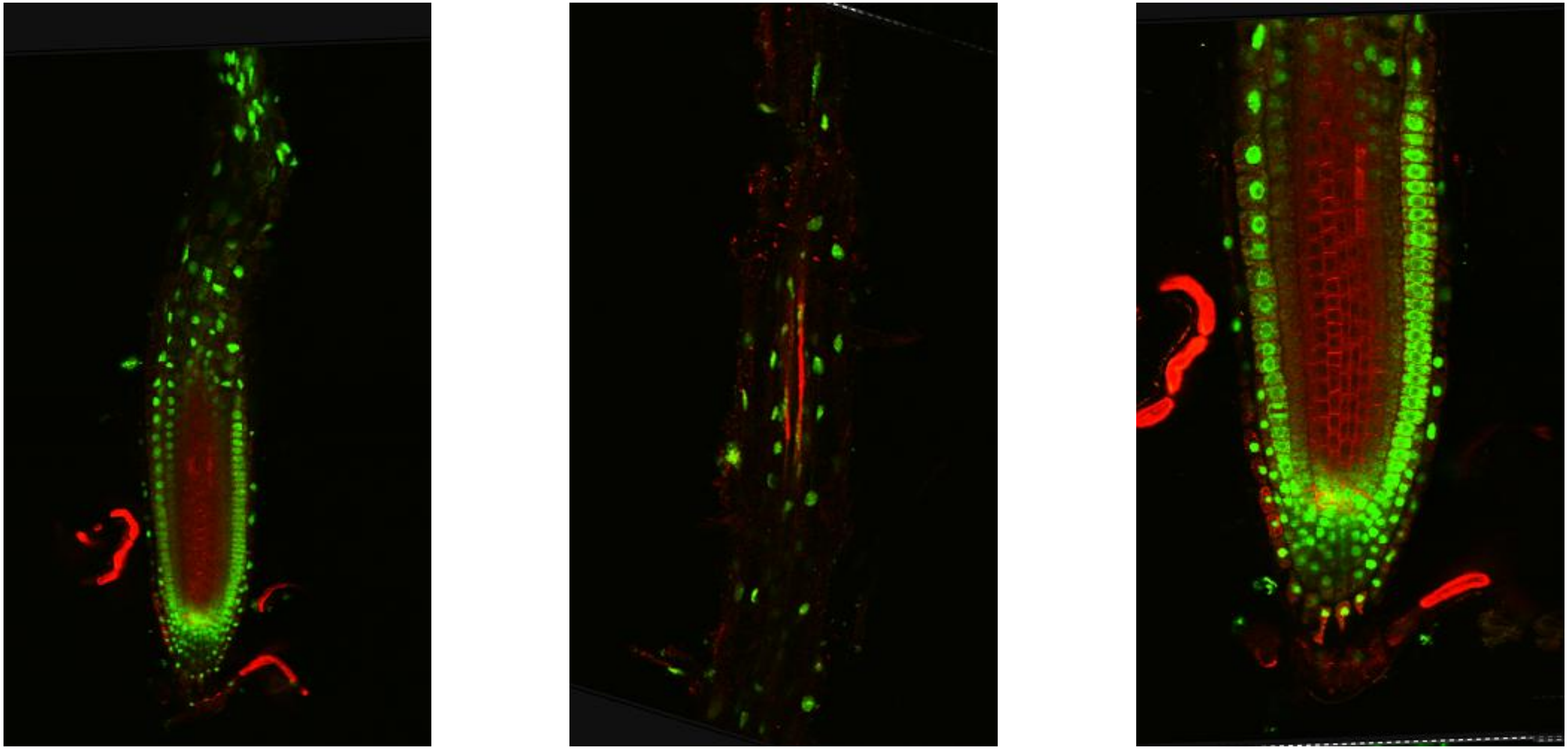

CCRC-M13

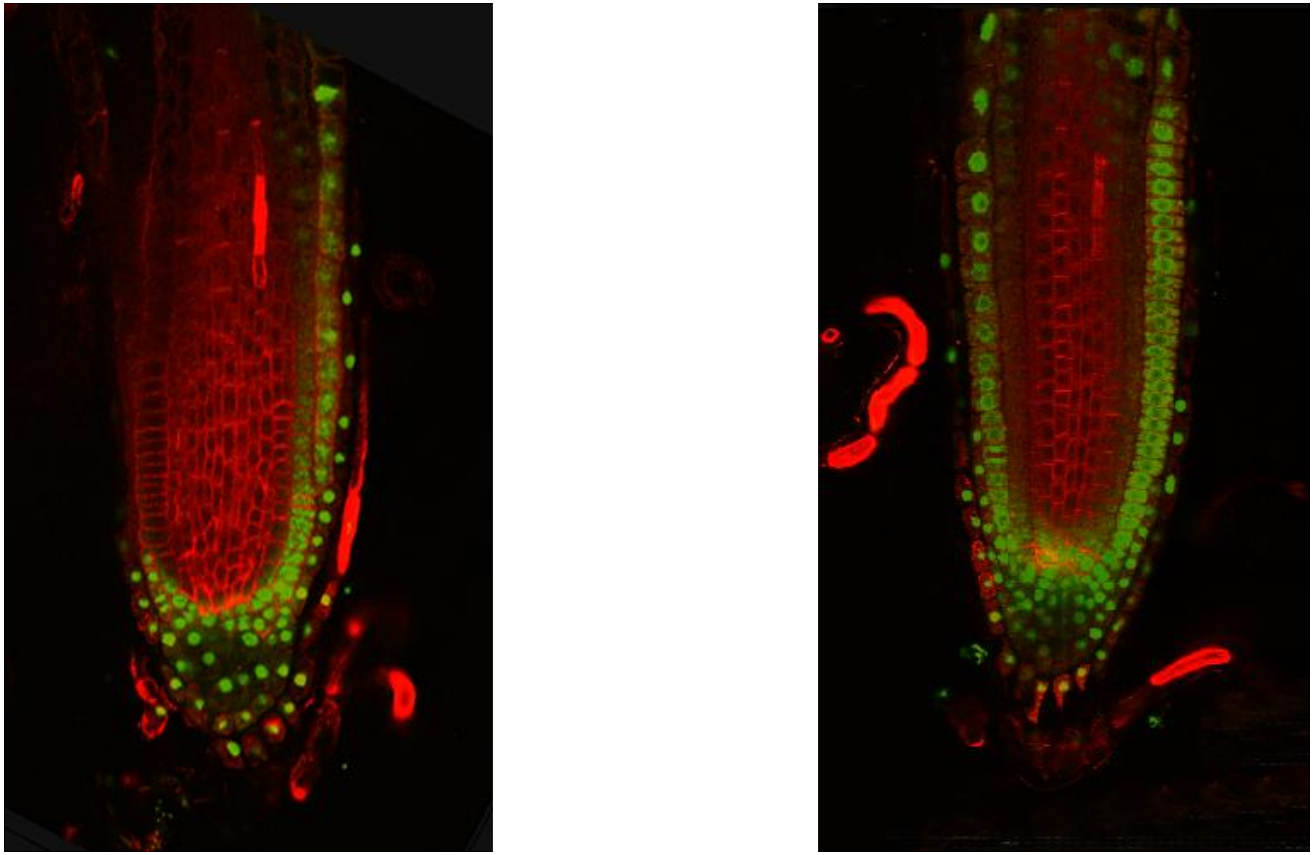

CCRC-M22

epitope not determined

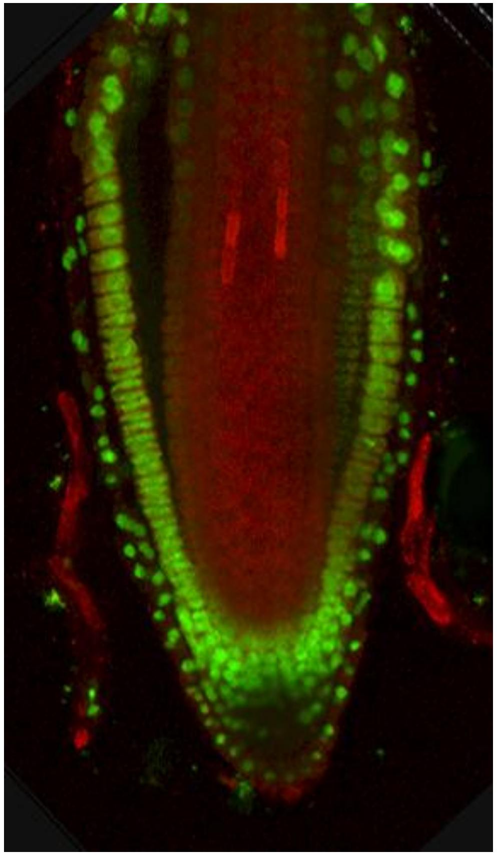

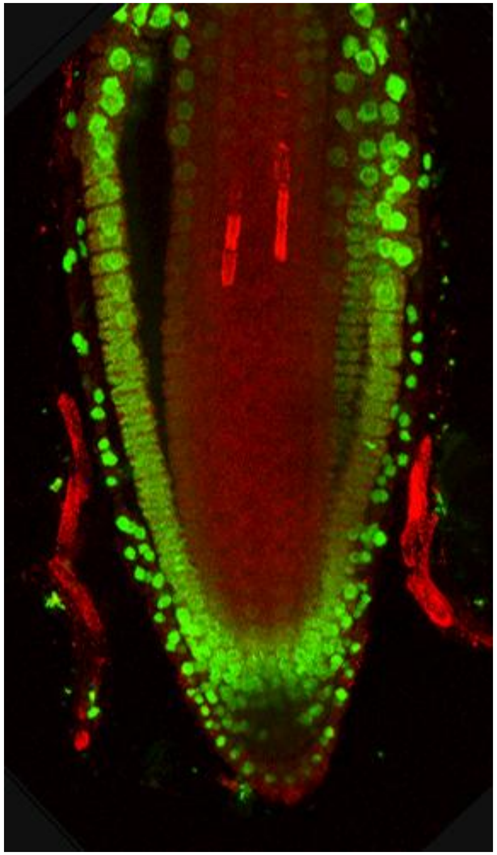

CCRC-M22

epitope not determined

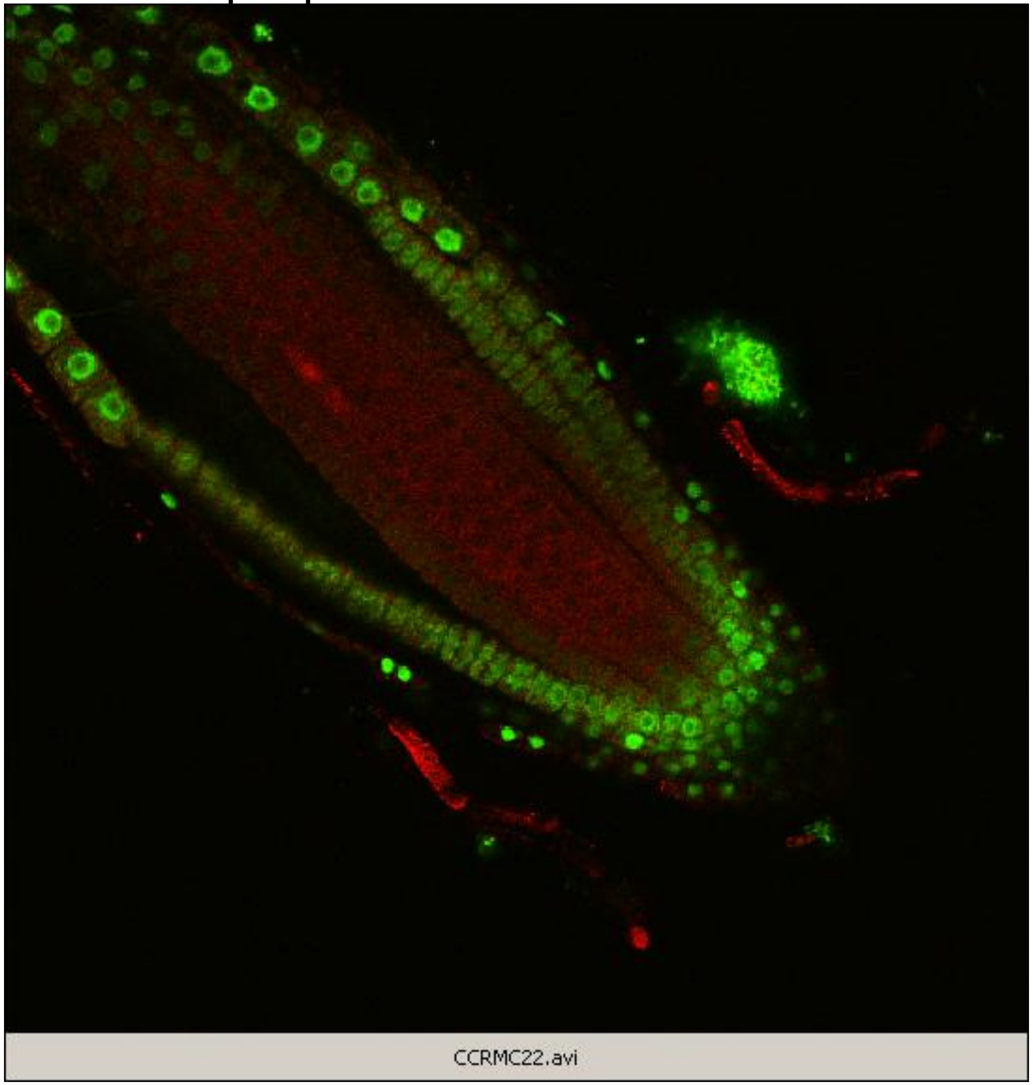

Supplement: Supplementary file 2 [file Image1.PDF]
