# Supplementary material for: Multi-omics analysis identifies genes mediating the extension of cell walls in the Arabidopsis thaliana root elongation zone
Source: Front Cell Dev Biol. 2015 Feb 20;3:10. doi: 10.3389/fcell.2015.00010 (PMC4335395; doi:10.3389/fcell.2015.00010)
Supplement: Supplementary file 3 [file Image2.PDF]

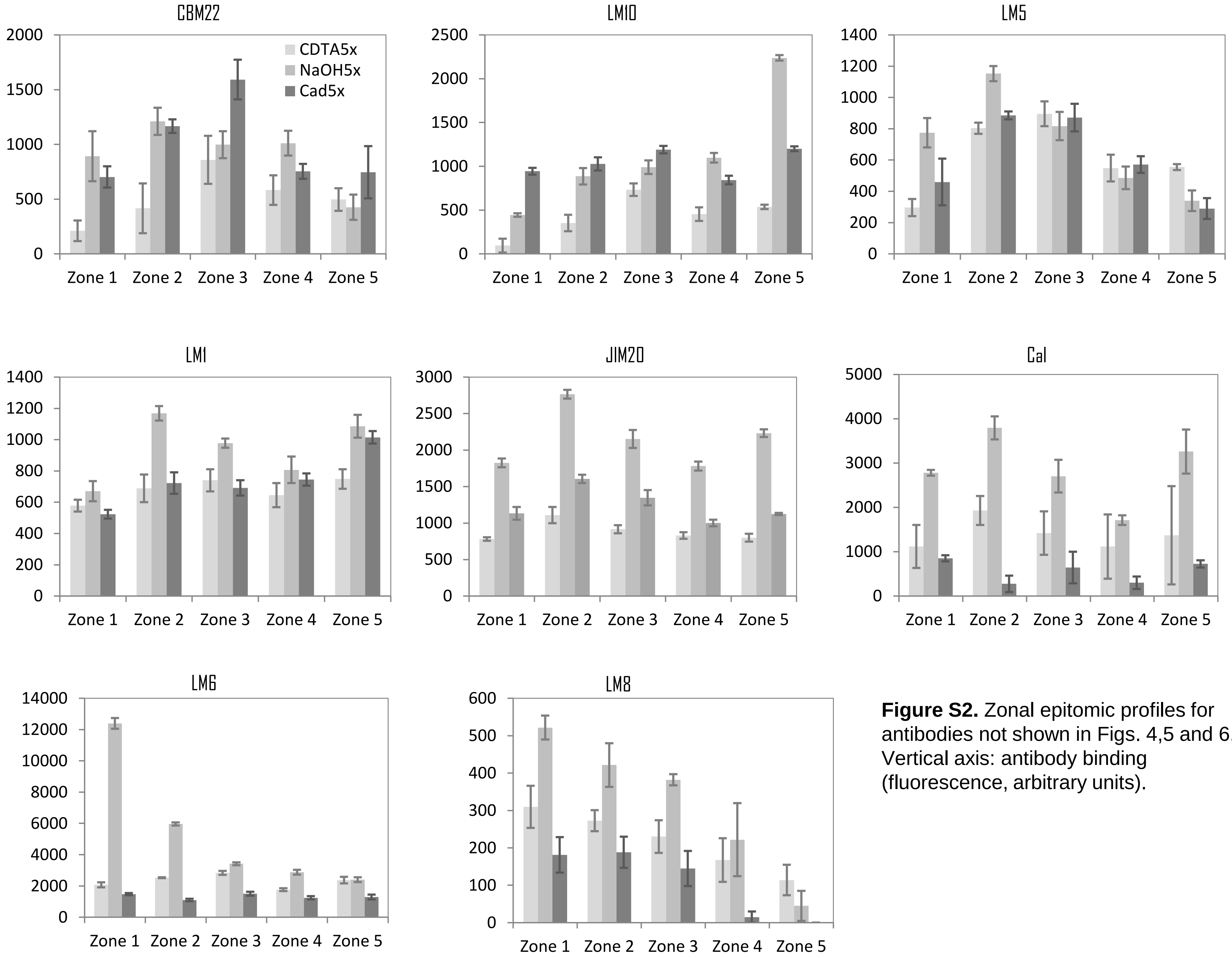

Figure S2. Zonal epitomic profiles for antibodies not shown in Figs. 4,5 and 6. Vertical axis: antibody binding (fluorescence, arbitrary units).
